# Increased Numbers of IL-7 Receptor Molecules on CD4+CD25−CD107a+ T-Cells in Patients with Autoimmune Diseases Affecting the Central Nervous System

**DOI:** 10.1371/journal.pone.0006534

**Published:** 2009-08-06

**Authors:** Nalini Kumar Vudattu, Sharon Kuhlmann-Berenzon, Mohsen Khademi, Vicki Seyfert, Thomas Olsson, Markus J. Maeurer

**Affiliations:** 1 Department of Microbiology, Tumor and Cell Biology (MTC), Karolinska Institutet, Stockholm, Sweden; 2 Smittskyddsinstitutet, Biostatistics and Epidemiological Modelling, Department of Epidemiology, Swedish Institute for Infectious Disease Control, Stockholm, Sweden; 3 Neuroimmunology Unit Department of Clinical Neuroscience, Karolinska Institutet at Karolinska University Hospital, Solna, Sweden; 4 Immune Tolerance Network, Department of Medicine, University of California San Francisco, San Francisco, California, United States of America; 5 Smittskyddsinstitutet, Section of Translational Immunology, Stockholm, Sweden; New York University School of Medicine, United States of America

## Abstract

**Background:**

High content immune profiling in peripheral blood may reflect immune aberrations associated with inflammation in multiple sclerosis (MS) and other autoimmune diseases affecting the central nervous system.

**Methods and Findings:**

Peripheral blood mononuclear cells from 46 patients with multiple sclerosis (MS), 9 patients diagnosed with relapsing remitting MS (RRMS), 13 with secondary progressive multiple sclerosis (SPMS), 9 with other neurological diseases (OND) and well as 15 healthy donors (HD) were analyzed by 12 color flow cytometry (TCRαβ, TCRγδ, CD4, CD8α, CD8β, CD45RA, CCR7, CD27, CD28, CD107a, CD127, CD14) in a cross-sectional study to identify variables significantly different between controls (HD) and patients (OND, RRMS, SPMS). We analyzed 187 individual immune cell subsets (percentages) and the density of the IL-7 receptor alpha chain (CD127) on 59 individual immune phenotypes using a monoclonal anti-IL-7R antibody (clone R34.34) coupled to a single APC molecule in combination with an APC-bead array. A non-parametric analysis of variance (Kruskal-Wallis test) was conducted in order to test for differences among the groups in each of the variables. To correct for the multiplicity problem, the FDR correction was applied on the p-values. We identified 19 variables for immune cell subsets (percentages) which allowed to segregate healthy individuals and individuals with CNS disorders. We did not observe differences in the relative percentage of IL-7R-positive immune cells in PBMCs. In contrast, we identified significant differences in IL-7 density, measured on a single cell level, in 2/59 variables: increased numbers of CD127 molecules on TCRαβ+CD4+CD25 (intermed) T-cells and on TCRαβ+CD4+CD25−CD107a+ T-cells (mean: 28376 Il-7R binding sites on cells from HD, 48515 in patients with RRMS, 38195 in patients with SPMS and 33692 IL-7 receptor binding sites on cells from patients with OND).

**Conclusion:**

These data show that immunophenotyping represents a powerful tool to differentiate healthy individuals from individuals suffering from neurological diseases and that the number of IL-7 receptor molecules on differentiated TCRαβ+CD4+CD25−CD107a+ T-cells, but not the percentage of IL-7R-positive cells, segregates healthy individuals from patients with neurological disorders.

## Introduction

Complex genetic traits and environmental factors [1] may contribute to immune responses associated with multiple sclerosis (MS), a demyelinating disease of the central nervous system characterized by a wide clinical variability [2] More recent studies addressed the question whether alterations in immune cell subsets in the peripheral circulation contribute to and reflect CNS inflammation in patients with MS [3]. MRI-active and MRI-inactive patients showed significant differences in the relative composition of several immune cell subsets, including TCRαβ+CD4+CCR7−CD45RA− peripheral memory T-cells [3] and a reduction of CD8low+CD56+ natural killer cells [4]. Profiling immune cell subsets in peripheral blood with an attempt to identify patterns associated with MS has been carried out since more than 25 years [5]. The advent of high content flow cytometric analysis enables now to look at a comprehensive number of immune cell subsets simultaneously at the single cell level. Most studies examined either the relative distribution or absolute numbers of immune cell subsets in PBMCs. In contrast, the number of biologically relevant molecules, e.g. CD127, the alpha chain of the IL-7 receptor, on single cells has not yet been addressed: IL-7, signaling through the IL-7 receptor, has been shown to increase antigen-specific T-cell responses in patients with MS [6] and provide long-term T-cell survival and thymic output [7], which may lead to the expansion of auto-reactive T-cell clones [8]. We examined in the current report the percentage of 187 individual T-cell subsets for association with IL-7R expression. This approach addresses differences in immune cell composition in peripheral blood mononuclear cells (PBMCs), but it does not measure differences of IL-7R expression on a single cell level. We measured therefore CD127 antibody binding sites on a more limited number of immune cell subsets (n = 59) to objectively define the number of IL-7R molecules on distinct T-cell phenotypes to identify cellular markers associated with MS.

## Results

### Cluster analysis of immune phenotypes segregates healthy blood donors from individuals with neurological diseases

**Figure 1:**
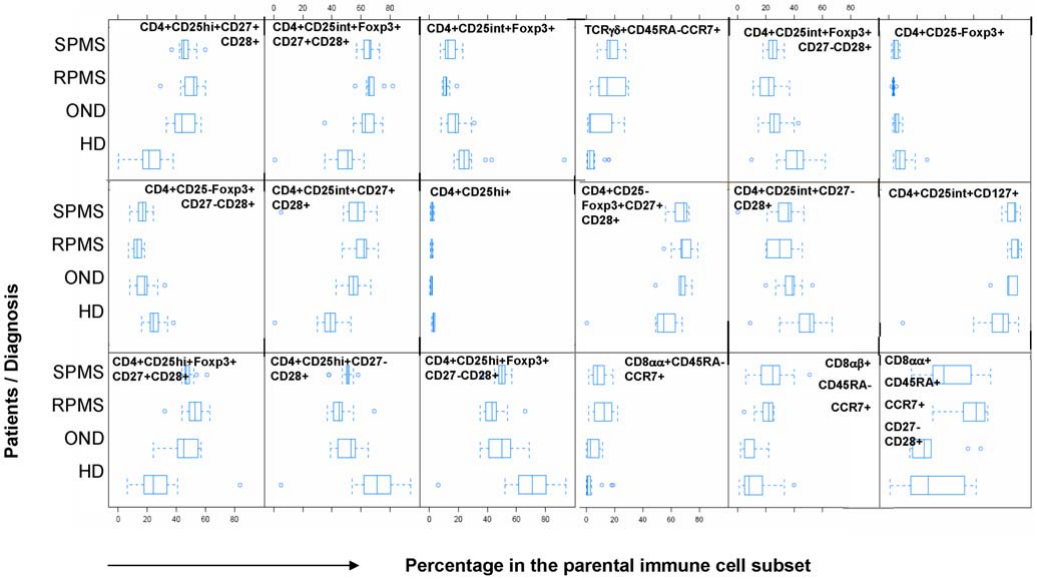
Boxplots of significant different frequencies of immune cells in healthy individuals versus patients with neurological diseases. Peripheral blood mononuclear cells from 46 patients with multiple sclerosis (MS), 9 patients diagnosed with relapsing remitting MS (RRMS), 13 with secondary progressive multiple sclerosis (SPMS), 9 with other neurological diseases (OND) and well as 15 healthy donors (HD) were analyzed (prior to therapy) by 12 color flow cytometry (TCRαβ, TCRγδ, CD4, CD8α, CD8β, CD45RA, CCR7, CD27, CD28, CD107a, CD127, CD14) in a cross-sectional study to identify variables significantly different between controls (HD) and patients (OND, RRMS, SPMS). We analyzed 187 individual immune cell subsets (percentages), 19/187 variables segregated PBMCs obtained from healthy individuals from individuals with neurological diseases. We show 18/19 variables, the number of CD127+CD19+ B-cells proved also to be different in HDs versus patients with autoimmune disease (data not shown).

187 individual phenotypes (see supplementary Table S1, i.e. S1.3) were measured to define the percentage of immune cell subsets in peripheral blood mononuclear cells (PBMCs) from healthy individuals and in patients with neurological disorders (Table 1), age and sex distribution for each individual group is provided in Table 2. A Kruskal-Wallis test was conducted and p-values were corrected using the FDR method. This resulted in 19 significant variables which differentiated healthy individuals and individuals with neurological disorders (Figure 1). We performed a heatmap analysis in order to visualize differences in the relative distribution of immune cell subsets in PBMCs obtained from healthy donors and from individuals with neurological diseases.

**Table 1:** Clinical profile of patient enrolled in the study.

Sample ID	Treatment	Sex	Age at sampling	Diag:G1	Diag:G2	Diag:G3	Diagnosis	EDSS	OB or Ig Index	MRI	MRI lesions
P1	No	F	31	MS	RR	RR-rem	RRMS-rem	1	Yes	Positive	9 or more
P2	No	F	35	CIS	CIS	CIS-relaps	CIS-Myelitis	3	Yes	Positive	9 or more
P3	No	F	30	MS	RR	RR-rem	RRMS-rem	2	Yes	Positive	3 to 5
P4	No	F	39	OND	OND	OND	Hypothyreosis and sensory disturbance	1	No	Normal	0
P5	No	F	25	OND	OND	OND	Paresthesia	0	No	Normal	0
P6	No	F	45	OND.INF	OND.INF	OND.INF	Neuroborreliosis	NA	No	Not done	NA
P7	Simvastatin Gabapentin Atenolol	F	67	MS	SP	SPMS	SPMS	3	Yes	Positive	9 or more
P8	No	F	39	OND.INF	OND.INF	OND.INF	Reccurent Myelitis	3	No	Normal	0
P9	No	F	66	OND.INF	OND.INF	OND.INF	Neuroinflammatory symptoms, potential side effects of earlier anti-TNF treatment	NA	Yes	Atypical	3 to 5
P10	Betaferon	F	36	MS	RR	RR-rem	RRMS rem	1	No	Atypical	9 or more
P11	No	F	43	OND	OND	OND	Paresthesia	0	No	Normal	0
P12	No	F	44	MS	RR	RR-rem	MS	1.5	No	Positive	9 or more
P13	No	M	27	OND	OND	OND	Parasthesia	1	No	Not done	NA
P14	Citalopram	F	34	OND	OND	OND	Carpal Tunnel Syndrome	NA	No	Normal	0
P15	No	F	55	CIS	CIS	CIS-rem	CIS Supratentorial	2.5	Yes	Positive	9 or more
P16	No	F	31	MS	RR	RR-rem	RRMS rem	0	LP not done	Positive	6 to 8
P17	No	M	59	MS	SP	SPMS	SPMS	3.5	Yes	NA	NA
P18	No	F	45	MS	SP	SPMS	SPMS	4	Yes	NA	NA
P19	No	M	51	MS	SP	SPMS	SPMS	5.5	Yes	NA	NA
P20	No	M	45	MS	SP	SPMS	SPMS	6	Yes	NA	NA
P21	No	F	60	MS	SP	SPMS	SPMS	4.5	Yes	NA	NA
P22	No	F	62	MS	SP	SPMS	SPMS	6	Yes	NA	NA
P23	No	F	57	MS	SP	SPMS	SPMS	5.5	Yes	NA	NA
P24	No	F	62	MS	SP	SPMS	SPMS	6	Yes	NA	NA
P25	No	M	54	MS	SP	SPMS	SPMS	6	Yes	NA	NA
P26	No	F	62	MS	SP	SPMS	SPMS	5.5	Yes	NA	NA
P27	No	M	43	MS	SP	SPMS	SPMS	4	Yes	NA	NA
P28	No	F	28	MS	RR	RR-rem	RRMS rem	1.5	Yes	Positive	9 or more
P29	No	M	25	MS	RR	RR-rem	RRMS rem	2	Yes	Positive	9 or more
P30	No	M	35	MS	RR	RR-rem	RRMS rem	2.5	Yes	Positive	9 or more
P31	No	F	44	MS	SP	SPMS	SPMS	3	Yes	Positive	9 or more

NA = not applicable, rem = remittent.

**Table 2:** Age and Sex ratios/group.

HC	Age	Sex	SPMS	Age	Sex	RRMS	Age	Sex	OND	Age	Sex
1	42	F	1	45	F	1	32	F	1	39	F
2	58	F	2	60	F	2	35	F	2	26	F
3	42	F	3	62	F	3	30	F	3	46	F
4	49	F	4	57	F	4	37	F	4	66	F
5	62	F	5	62	F	5	55	F	5	44	F
6	51	M	6	62	F	6	28	F	6	43	F
7	24	M	7	44	F	7	31	F	7	39	F
8	58	M	8	68	F	8	25	M	8	34	F
9	51	M	9	43	M	9	35	M	9	27	M
10	48	M	10	54	M	median	32		median	39	
11	58	M	11	51	M						
12	NA		12	59	M						
13	NA		13	45	M						
14	NA		median	57							
15	NA										

HC - Healthy Control.

SPMS - Secondary Progressive Multiple Sclerosis.

RRMS - Relapse and Remittent Multiple Sclerosis.

OND - Other Neurological Diseases.

F - Female.

M - Male.

**Figure 2:**
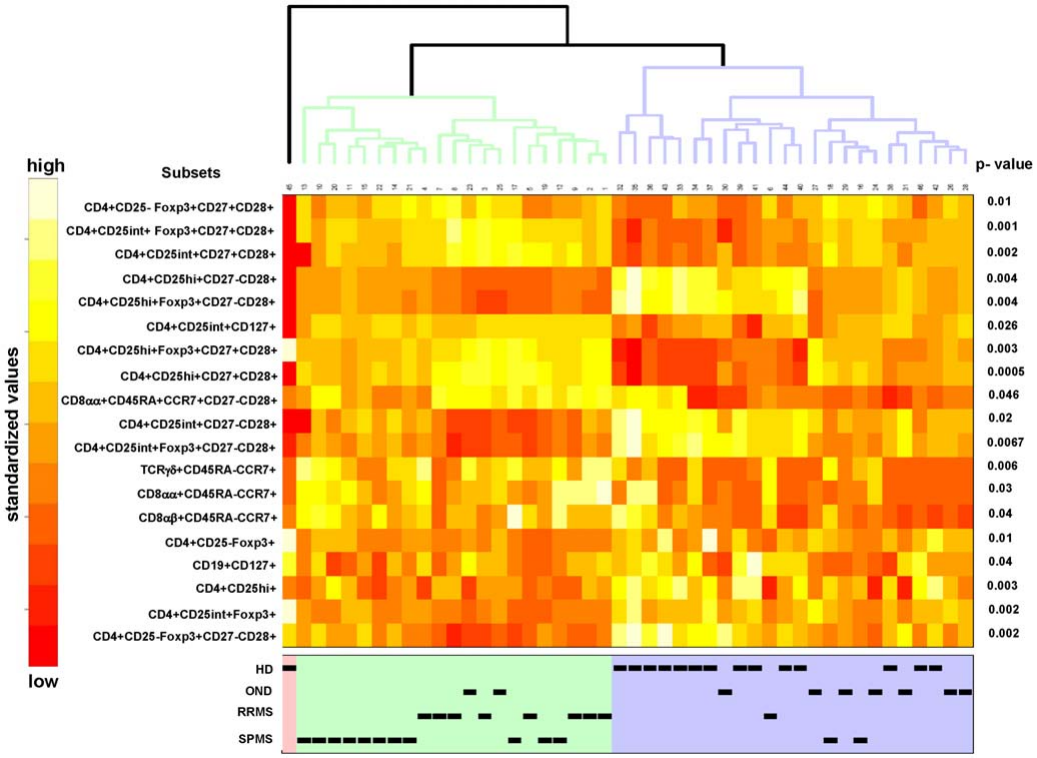
Visualization of differences in immune cell frequencies using heatmaps. After quality data extraction and statistical analysis (Kruskal-Wallis test, see material and methods), p-values were obtained and a cutoff of 0.05 was applied to obtain a subset of significant variables. The final analysis included a principal component analysis (PCA) to study the correlation between the variables, and cluster analysis to see the connection between variables and groups of patients. Lighter colors (white) represent higher standardized values and darker represent lower standardized values (red) summarizing cellular immune phenotypes (percentages of immune cell subsets). The individual 45 (a healthy blood donor is included in the analysis). This individual showed aberrant immune markers in PBMCs.

**Figure 3:**
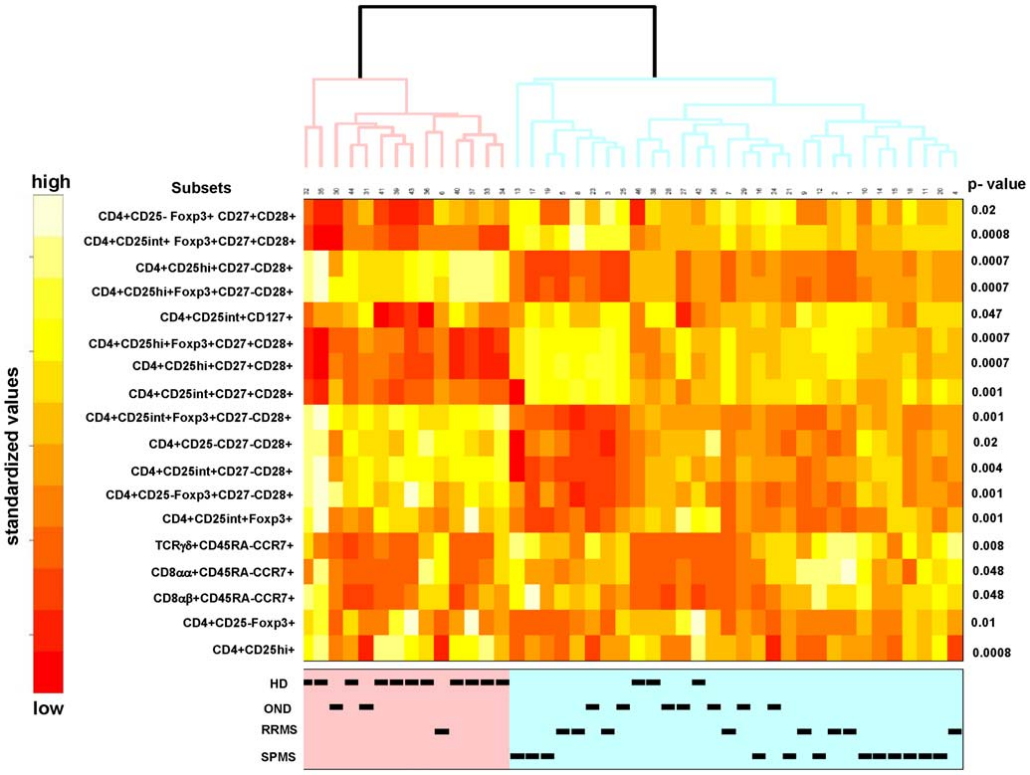
Visualization of differences in immune cell frequencies using heatmaps. Similar data analysis as in Figure 3, yet data from individual 45 were excluded. This allowed to cluster immune markers obtained in PMBCs from healthy individuals and individuals with inflammatory processes of the central nervous system. Lighter colors (white) represent higher standardized values and darker lower standardized values (red) summarizing immune phenotypes. p values are provided (right).

Analysis of PBMCs obtained from individual number 45, a healthy donor, showed a very different pattern of immune cell subsets as compared to the rest of the individuals. The statistical testing and multiplicity correction were therefore run in two different variants: including (Figure 2) and excluding individual number 45 (Figure 3) from the statistical analysis. The significant 19 variables are listed with the corresponding p-values in heatmaps, each color represents a defined percentage of the target immune cell population in lymphocytes. It is evident that all phenotypic variables (percentages of immune cell subsets) are slightly different in each individual. In general, clustering the 19 different variables showed that phenotypic markers, defined by flow cytometry, allow to segregate healthy individuals from individuals with neurological diseases. This exercise also confirmed the results that individual # 45 (a healthy blood donor) exhibited a different phenotypic pattern in PBMCs (Figures 2 and 3), clinically very well defined populations are crucial for the identification of markers which allow to identify an individual as ‘healthy’ or suffering from a (inflammatory, neurological) disease. The heatmap analysis also indicated that certain immunophenotype subgroups discern between HD and OND groups and patients with MS (RRMS and SPMS). In general, two groups were formed: healthy donors on one side, and OND, RRMS, and SPMS on the other. Differences could be identified in the relative percentages of Treg cells, including CD3+CD4+/CD25^high^+/Foxp3+/CD27+CD28+ T-cells; increased percentages could also be identified in TCRγδ+/CD45RA−CCR7+ T-cells, in TCRαβ+/CD8αα+/CD45RA−CCR7+ T-cells as well as in the TCRαβ+/CD8αα+/CD45RA+CCR7+/CD27−CD28+ T-cell subset.

### Differences in IL-7R density on immune cell subsets

**Figure 4:**
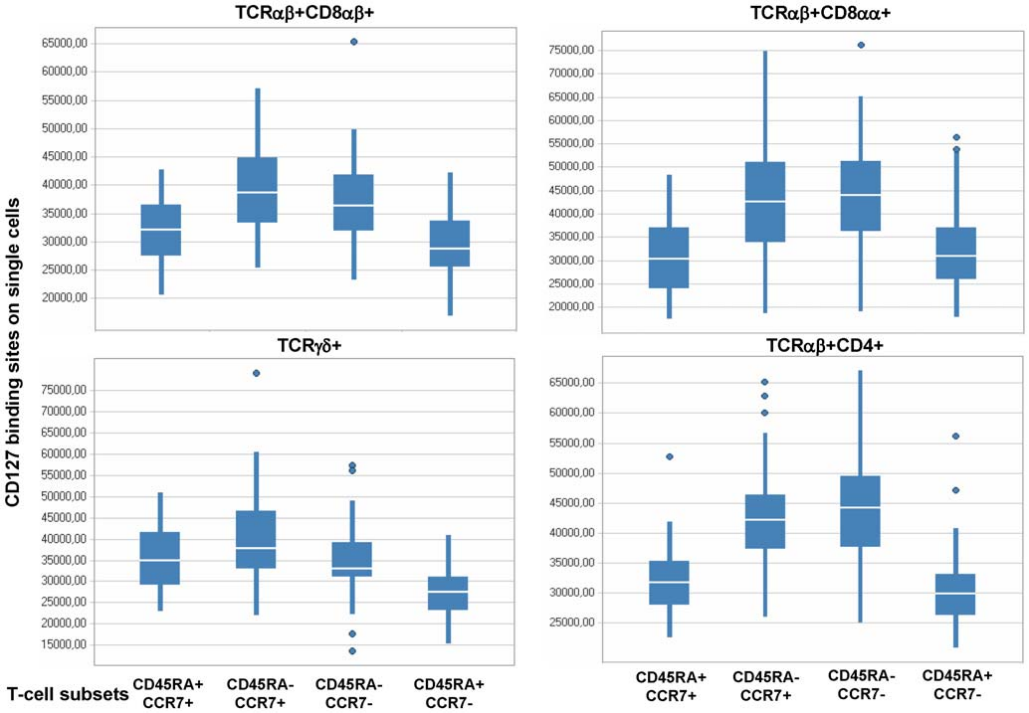
IL-7 receptor density is associated with T-cell maturation. IL-7 receptor molecules were enumerated on the single cell level in PBMCs from healthy controls and from patients with neurological disorders. We could not identify significant differences in IL-7R expression in TCRαβ, CD8αβ, CD8αα, TCRγδ or on TCRαβ+CD4+ T-cell subsets, therefore we show the data from all individuals enrolled in the study. T-cells with the CD45RA−CCR7+ phenotype exhibit the highest density of IL-7R molecules, followed by CD45RA−CCR7− T-cells and CD45RA+CCR7+(precursor) T-cells. Differentiated CD45RA+CCR7− exhibit the lowest number of IL-7R molecules. Mean and median for each T-cell subset clustered by patient groups is provided in the supplementary Table S2. CD127 expression appears to be associated with T-cell maturation/differentiation.

**Figure 5:**
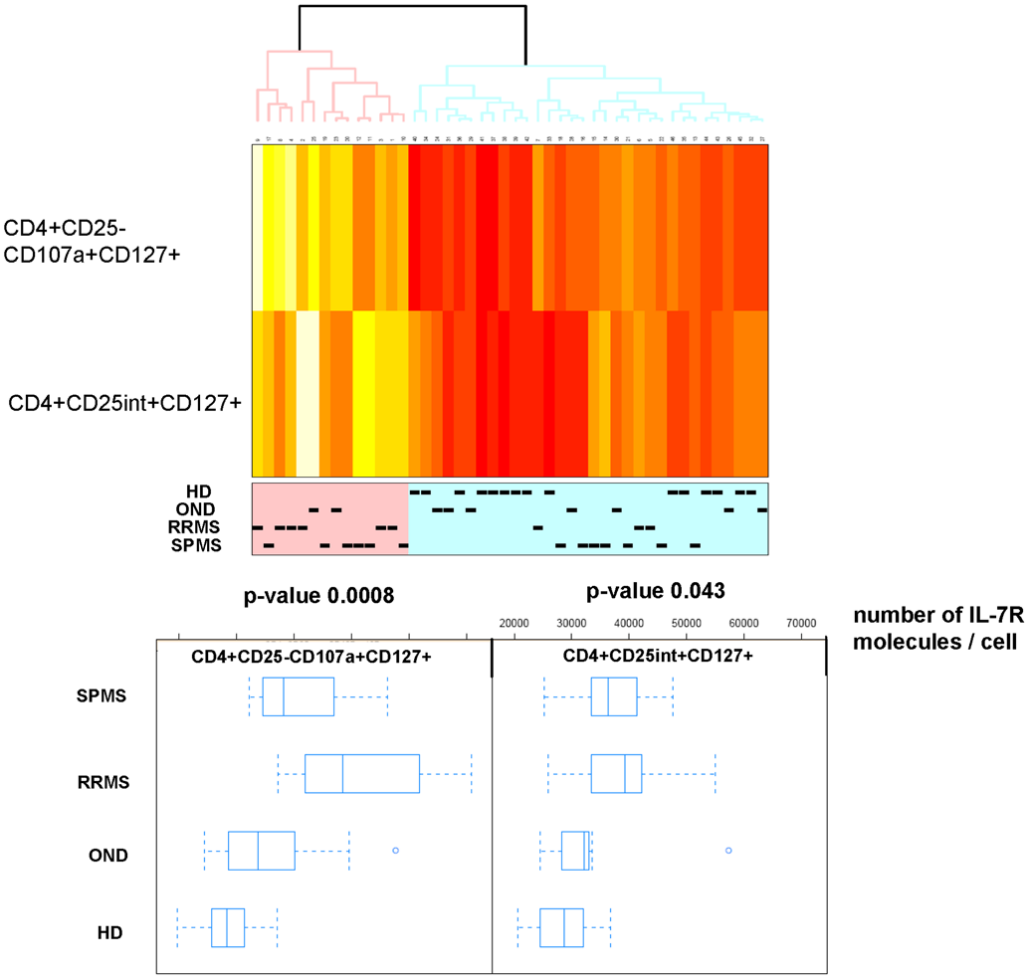
Significant differences in IL-7R densities in immune cell subsets. IL-7 receptor (CD127) density analysis was carried in 59 individual immune phenotypes using a monoclonal anti-Il-7R antibody (clone R34.34) coupled to a single APC molecule in combination with an APC-bead array. A non-parametric analysis of variance (Kruskal-Wallis test) was conducted in order to test for differences among the patient groups for each of the variables. 2/59 variables showed increased numbers of IL-7R molecules on TCRαβ+CD4+CD25(intermed)+ T-cells and on TCRαβ+CD4+CD25−CD107a+ T-cells (mean: 28376 Il-7R binding sites on cells from HD, 48515 in patients with RRMS, 38195 in patients with SPMS and 33692 IL-7 receptor binding sites on cells from patients with OND). Differences are visualized using a heatmap (top panel) and boxplots (bottom panel). p values are provided.

Although we identified 19 significantly different immune markers in PBMCs which allowed to segregate healthy individuals and individuals with neurological disease, none of these markers were associated with expression of the Il-7 receptor. The analysis addressed only the percentage of Il-7R-positive T-cells in lymphocytes, it reflected the relative composition of IL-7R+ cells in PBMCs. Differences in immune cell subsets, as well as biological responses may not only be associated with the relative distribution of immune cells, but also with the absolute number of (IL-7R) molecules on single cells. We extended therefore the analysis of cellular immune abnormalities associated with CNS inflammation to the number of Il-7R molecules on the single cell level. 59 immune cell subsets (listed in the supplementary Table S1 online, S1.4) were examined for differences in IL-7 receptor densities. IL-7R density was associated with physiological alterations in immune cell subsets reflecting T-cell maturation/differentiation. Differentiated T-cells, defined by the CD45RA+CCR7− phenotype, exhibited the lowest number of IL-7 receptor molecules (Figure 4) and central/peripheral memory T-cells (CD45RA−CCR7+ or CD45RA−CCR7−) exhibited the highest IL-7R density. Mean, median values and standard deviation for the enumeration of Il-7R molecules are provided for each of the 59 individual immune cell subsets, segregated for each patient group and healthy donors (Supplementary Table S2). We could not identify statistical significant differences within these T-cell subsets, defined by CD45RA and CCR7 expression, which segregate healthy individuals and individuals with neurological diseases. However, we could identify two immune cell subsets which showed significant differences in IL-7R density on the single cell level. A clear difference among PBMCs from healthy blood donors and individuals with neurological diseases can be seen in the boxplots of TCRαβ+CD4+CD25−CD107a+CD127+ T-cells (p = 0.0008) and less clearly in TCRαβ+CD4+CD25^int^+CD127+(p = 0.043) (Figure 5), the absolute number of events for each T-cell population segregated by groups is provided in the supplementary Table S3. With these two variables, the clustering of individuals provided a rather good discrimination of PBMCs from healthy individuals, less clear discrimination of OND and RRMS, and none at all of SPMS based on heatmap cluster analysis (data not shown). PBMCs from healthy donors showed 28664 (mean value) IL-7R molecules on TCRαβ+CD4+CD25−CD107a+CD127+ T-cells, 50734 (mean value) molecules were observed on the same T-cell subset in PBMCs from patients with RRMS, 40634 (mean value) molecules were identified on TCRαβ+CD4+CD25−CD107a+CD127+ T-cells from individuals with SPMS and 36331 (mean value) molecules were enumerated on this T-cell subsets in PBMCs from patients with OND.

## Discussion

Among 187 individual immune cell phenotypes, only a few turned out to be statistically different in patients with MS as compared to healthy controls. High content flow cytometric analysis allows to analyze a plenitude of immune cell markers visualizing changes in distinct immune cell subsets. Only the combination of appropriate cell surface makers enabled us to define differences in distinct immune cell subsets. For instance, a population of of CD8^low^+CD4− cells was recently identified in untreated RRMS patients, as well as in individuals with a clinically isolated demyelation syndrome; these differences had been attributed to a reduction in CD8^low^+CD56+CD3− natural killer cells [4]. A more detailed prospective study in twenty patients who were observed longitudinally [3] showed significant differences in ten lymphocyte subsets associated with active MS and these markers included the innate and the adaptive arms, i.e. TCRαβ+CD4+CCR7−CD45RA− T-cells, TCRγδ+CCR5+ T-cells and regulatory T-cells. If we exclude individual 45 (Figure 3) which showed a very similar profile as compared to individuals with neurological diseases, we could also identify reduced numbers of TCRαβ+CD4+CD25^high+^ T-cells (p = 0.0008). The simultaneous analysis of multiple markers (i.e. TCRαβ, CD25, Foxp3, CD27, CD28 and CD127) for Treg cells showed a relative increase of TCRαβ+CD4+CD25^high^+CD27+CD28+ T-cells within the CD4+CD25^high^+ T-cell population, as well as an increase of CD3+CD4+CD25^int^+Foxp3+CD27+CD28+ T-cells. These CD4+ T-cell subsets suggest an enrichment of activated T-cells with the CD27+,CD28+ phenotype since Foxp3 expression is not only a marker of Treg cells, it also indicates T-cells activation: IL-2, IL-15, as well as IL-7 are able to transiently induce Foxp3 in CD4+CD25− effector T-cells [9].

The reduction of CD4+CD25^high^ T-cells was due to a reduction in TCRαβ+CD4+CD25^high+^ CD27−CD28+ as well as in CD3+CD4+CD25^high^+Foxp3+CD27−CD28+ T-cells. Of note, the Treg subsets, which showed to be decreased in patients with MS as compared to healthy individuals, expressed CD28 and some Treg subsets expressed invariably CD27 (Figures 2 and 3) consistent with the notion that CD28 engagement controls homeostasis of CD4+CD25+ Treg cells [10]. A decrease of Treg cells in the peripheral circulation has been described in patients with MS [11], and in other autoimmune diseases, i.e. RA [12]. Natural regulatory T-cells express CD25 which resulted in the differentiation of regulatory (CD25high), activated (CD25intermed) and ‘precursor’ CD4+CD25^low^ T-cells [13]. We did not observe significant differences between healthy donors or patients with neurological diseases in IL-7R (CD127+) frequency or receptor density on Treg cells. Low CD127 expression, along with high CD25 expression has been shown to better define Treg cells as compared to T-cells which express FOXP3+: CD4+CD25^high+^CD127^low+^ T-cells show highly suppressive activity [14], [15]. Expression of CD127 is negatively associated with FOXP3 due to promoter-interaction [16]. However, there is a minor, yet detectable population in CD4+CD25^high+^CD127+ cells, these T-cells showed higher proliferative capacity and produced more IFNγ and IL-2 as compared to cells from healthy control individuals [17] suggesting an abnormal function of CD4+CD25^high^, CD127+ T-cells in patients with MS.

We observed an increase in the percentage of central memory TCRαβ (CD45RA−CCR7+) in CD8αβ+ and CD8αα+ T-cell subsets. The latter immune cell population presents a distinct memory T-cell subset, presumably associated with long-term antigen exposure. CD8αα+ T-cells have been shown to be present in chronic infections [18] and they are enriched in antigen specific T-cell responses in patients with cancer [19] or in nonhuman primates after BCG vaccination [20]. The percentage of central memory phenotype CD45RA−CCR7− cells was also statistically significant increased in TCRγδ+ T-cells in patients with MS, consistent with earlier reports which showed increased TCRγδ+ T-cells in target tissues [21]. CD8+ T-cells may show detrimental immune effector functions and contribute to chronic inflammation [22], yet they may also exert suppressive functions [23]. Noteworthy, anti.-CD25 included in our panel, or other T-cell activation or differentiation makers, e.g. CD27 or CD28 did not segregate CD8+ T-cell subsets in patients from MS versus healthy individuals. Only the combinatory analysis of the CD8α and CD8β chain along with CD45RA, CCR7+, CD27 and CD28 marker analysis was able to segregate CD8 phenotypes in patients with MS from CD8+T-cells from healthy blood donors.

Although the IL-7R was recently incriminated as a marker for increased risk to develop MS [24], [25], we could not identify the IL-7R in the analysis of immune cell subsets as a discriminatory marker between healthy individuals and patients with MS. Most flow cytometric analyses measure the percentage of distinct immune cell subset in the parental cell population. However, functional differences in immune cells could also be associated with the density of certain receptor molecules on the single cell level. Therefore, we measured the absolute number of IL-7 receptor molecules on the single cell level on 59 individual immune cell subsets (see online supplementary Table S2). The number of IL-7R molecules/cells showed a range between 30.000 to 50.000 molecules/cell in distinct immune cell subsets, the range is dependent on T-cell maturation (Figure 4). Memory T-cells show a higher IL-7R density as compared to effector T-cells (see details in supplementary Table S2). Only 2/59 immune cell phenotypes showed significant differences in the comparison of PBMCs from healthy individuals and patients with neurological diseases: TCRαβ+CD4+CD25−CD107a+ T-cells expressed higher numbers of IL-7R molecules as compared to PBMCs obtained from control individuals. Most likely, CD4+CD25− T-cells may represent terminally differentiated CD4+ T-cells [9]. Although CD25 has not been examined, a similar CD4+ T-cell phenotype was reported for mature, human CMV-specific effector CD4+ T-cells: these T-cells produce MIP-1β, TNFα and IFNγ in the absence of IL-2 and exhibit lytic activity associated with perforin and granzyme expression [26]. Analysis of CD107a and CD127 in PBMCs from patients with breast cancer showed a trend, but not statistically solid differences, towards lower values of IL-7R expression on CD4+CD107a+ T-cells [27]. We could show that CD4+ T-cells obtained from patients with breast cancer exhibit a reduced responsiveness to IL-7, defined by statistically reduced STAT-5 phosphorylation; these data suggested that differences in IL-7R expression, on the single cell level, may indeed translate into biologically relevant responses to IL-7 [27]. Future functional studies which link IL-7–mediated cellular downstream events to IL-7R density will aid to study the clinical significance of increased Il-7R expression. Since CD4+CD25− T-cells represent terminally differentiated (effector) T-cells, we postulate that chronic antigenic exposure and prolonged inflammation may contribute to the expansion of this subset in patients with MS. It is important to note that CD107a positive T-cells show increased IL-7R numbers on the single cell level (Figure 5). It remains to be shown whether co-expression of CD107a and CD127 reflects a general feature of T-cells with effector granules, or if the CD107a+CD127+ T-cell phenotype represents a CD4+ T-cell population associated with autoimmune diseases or chronic inflammatory processes: CD4+CD25− cells from patients with SLE were shown to be less sensitive to Treg activity as compared to T-cells from healthy controls [28]. and a more recent study showed increased numbers of CD4+CD25−Foxp3+ T-cells in patients with new-onset lupus erythematosus [29]. We propose that the TCRαβ+CD4+CD25−CD107a+ T-cell subset, defined by increased numbers of IL-7R molecules, represents a terminally differentiated [9] CD4+ T-cell population with effector granules, associated with chronic exposure to antigen stimulation.

## Materials and Methods

### Immune cell phenotyping

Blood was obtained from healthy, age and sex-matched blood donors and from individuals with neurological diseases (listed in detailed in Table 1 and 2) after informed consent (Diary number 02-548, dated 20.Nov.2002, ethical committee Stockholm). PBMCs were isolated using Ficoll, frozen in 90% fetal calf serum and 10% DMSO until analysis. The recovery was 89–97% and live lymphocytes were gated on forwarded versus sidescatter plot using flow cytometry. Three different mAb panels (supplementary Table S1.1 and S1.2) were used to define immune cell subsets listed in the supplementary Table S1 (S1.3 and S1.4). The antibody panel has been and titrated and evaluated by testing each mAb alone, or in combination with a mix of 11 corresponding monoclonal reagents, then by simultaneous incubation with 2 antibodies, followed by incubation with 10 reagents. The next step included testing of a mix of 3 mAbs, followed by staining with 9 mAbs. This approach resulted in the following protocol: 0.5 million PBMCs were first stained with anti-CCR7 for 15 minutes at 4°C, followed by addition of the 10 color antibody mix (designated as panel 1 and panel 2, see Table) and incubated for 15 minutes at 4°C. The anti-CD27 antibody was then added to cells which were incubated at 4°C, for 15 minutes, followed by washing with 1 ml of PBS containing 0.1% BSA. The cell pellet was resuspended in 200 ul of PBS (0.1%BSA). The first antibody panel (listed in the supplementary Table S1, S1.2) was used to define 53 individual immune cell subset (S1.3) and panel 2 defined 86 subsets (S1.3) The third panel (supplementary Table S1.2) was used to identify Treg cells yielding 44 individual immune cell subsets: PBMCs were stained with cell surface marker antibodies (panel 3) and incubated at 4°C for 15 minutes. PBMCs were then washed immediately with FACS staining buffer (BD Pharmingen, San Diego, USA) and fixed with 1 ml of 1× Fix/Perm buffer (Biolegend, San Diego, CA, USA ) at room temperature for 20 minutes followed by one washing step with FACS staining buffer and one time with 1× Permeabilization buffer (Biolegend). PBMCs were resuspended in 1 ml of 1× permeabilization buffer and incubated at room temperature for 15 minutes, followed by centrifugation at 250 g for 5 minutes. The cell pellet was resuspended in 100 ul of 1× permeabilization buffer and the Alexa 488-conjugated anti-Foxp3 antibody was added, followed by an incubation at room temperature in the dark for 30 minutes and washed, resuspended the pellet in 100 ul of staining buffer. Flow cytometric analysis was performed using a FACS Aria (BD Biosciences, Immunocytometry, San Jose, USA) and data were analyzed using FACS DIVA software (BD Biosciences). Results were reported in an excel spreadsheets as percentage or number of CD127-binding sites on individual immune cell subsets. Sequential gating strategies are depicted, as a paradigm, in the supplementary Figure S1. Freshly harvested PBMCs and the corresponding frozen/thawed aliquots from three healthy blood donors were analyzed for immune cell marker expression. Similar data were obtained (data not shown) which corroborates that the flow cytometric data are relevant for frozen as well as for freshly harvested PBMCs. Note that the percentage of immune cells relating to frequency in the parental population, is indicated with a slash, i.e. TCRαβ+/CD4+CD25hi+/CD27+CD28+ or TCRαβ+/CD4+CD25int+/Foxp3+/CD27+CD28+ cells.

IL-7R binding site analysis on a single cell level. The former analyses defined percentages in individual immune cell subsets. In order to define the absolute number of IL-7 receptor molecules on immune cell subsets on a single cell level, we obtained a preparation of the anti-IL-7 receptor alpha chain monoclonal mAb (clone R34.34) labeled with a single APC molecule. This allows to determine antibody binding sites, since the APC fluorescence intensity can be compared to the fluorescence intensity associated with the defined number of APC molecules on beads (supplementary Figure S2). Blank beads were suspended in 0.5 ml of PBS and instrument settings were adjusted using ‘blank beads’, followed by addition of beads (Bangs Laboratories, Inc. Indianapolis, USA) with three different APC fluorescent intensities which reflect the number of molecules/bead, 10e5 events were aquired. One drop of blank microspheres was resuspended in 0.5 ml of PBS and the flow rate was adjusted to 100–200 beads per second. The live gate of singlets was created on a forward versus sideward scatter dot plot and a APC histogram which showed only cells within the live gate. APC-beads with three different fluorescence intensities (reflecting the number of APC molecules/beads) were added to the tube with blank beads. A calibration plot was established using Quickcal software (Bangs Laboratories, Inc. USA ) using the median channel fluorescence intensity values for each bead population loaded with a distinct number of APC molecules. All PBMC samples were run with the same instrumentation settings. Antibody binding capacity values per cell were determined using the median value of CD127 (in APC molecules) of each immune cell subset using Quick Cal soft using the beads-calibration plot.

### Data management and data analysis

Data were originally compiled in excel files with different sheets containing data for the variables tested. Each patient had a unique identification code and the corresponding disease group. Analyses were conducted separately for the immune phenotypes (percentages for immune subsets) and the Il-7R density analysis. Quality control of the data was performed by summary statistics to detected anomalies in the data sets. Each individual variable was tested for the null hypothesis of ‘no difference’ among the disease groups using the non-parametric Kruskal-Wallis test. The alternative hypothesis is that at least one disease group differed from the other disease group (or the healthy control group). We choose a non-parametric approach due to the small number of individuals in each group. Given the limited data and thus low statistical power, no further tests to identify the deviating group (or groups) were conducted. Since a large number of tested were conducted, i.e. multiple comparisons, we applied a method that controls for false discovery rate (FDR) [30] and obtained new adjusted p-values for each variable. The significance level was set to 0.05. To visualize the results, boxplots for the significant variables were drawn. We clustered the individuals based on their values of the significant variables. The clustering algorithm was an agglomerative hierarchical method based on Euclidean distance of the average [31]. Heatmaps were produced to visualize the clustering results (Alexander Ploner, unpublished: Heatplus: A heatmap displaying covariates and coloring clusters, R package version 1.6.0). The heatmaps displayed the flow cytometric markers (in percentage of the parental T-cell population) for each individual patient. First, the top and bottom 1 percent of the values were trimmed to avoid extreme values, and then normalized for each marker by subtracting the mean and divided by the standard deviation. The values were represented on a 12-step equidistant scale, lighter colors (white) represent higher standardized values and darker colors (red) represent lower standardized values. All analyses were carried out in the statistical language R, version 2.6.2. (http://www.R-project.org)

## Supporting Information

Figure S1 Detailed gating strategies for T-cell subsets, including Tregs.
(0.07 MB PDF)

Figure S2 Paradigm of IL-7R (CD127) enumeration on immune cells. A. Cells from the lymphogate were subjected to further gating (n = 59 different subsets). The anti-IL-7 receptor alpha chain monoclonal mAb (clone R34.34) labeled with a single APC molecule was used to define CD127 antibody binding sites, since the APC fluorescence intensity can be compared to the fluorescence intensity associated with the defined number of APC molecules on beads (middle panel) with different APC molecules (top panel, right). A calibration plot was established using Quickcal software V2.3 (Bangs Laboratories, Inc. USA) using the median channel fluorescence intensity values for each bead population loaded with a distinct number of APC molecules. All PBMC samples were run with the same instrumentation settings. Antibody binding capacity values per cell were determined using the median value of CD127 (in APC) of each immune cell subset using Quick Cal soft using the beads calibration plot. B. Although the number of CD127+ T-cells may be different, e.g. lower in individuals with autoimmune disease, the expression of CD127 molecules on single cells may be higher.
(0.05 MB PDF)

Table S1 Compilation of monoclonal antibodies and immune cell subsets.
(0.03 MB PDF)

Table S2 Mean and Median values of IL-7 Receptor densities on T-cell subsets
(0.08 MB PDF)

Table S3 Number of events (min-max) for TCRαβ+CD4+CD25−CD107a+CD127+ and TCRαβ+CD4+CD25intermed+CD127+ T-cell subsets
(0.02 MB PDF)
