# Supplementary material for: Increased Numbers of IL-7 Receptor Molecules on CD4+CD25−CD107a+ T-Cells in Patients with Autoimmune Diseases Affecting the Central Nervous System
Source: PLoS One. 2009 Aug 6;4(8):e6534. doi: 10.1371/journal.pone.0006534 (PMC2717329; doi:10.1371/journal.pone.0006534)

Supplementary Figure(s) S1: Detailed gating strategies

G 1 Gating strategy for CD45RA-CCR7+ subsets from TCRαβ, TCRαα and TCRγδ T-cells

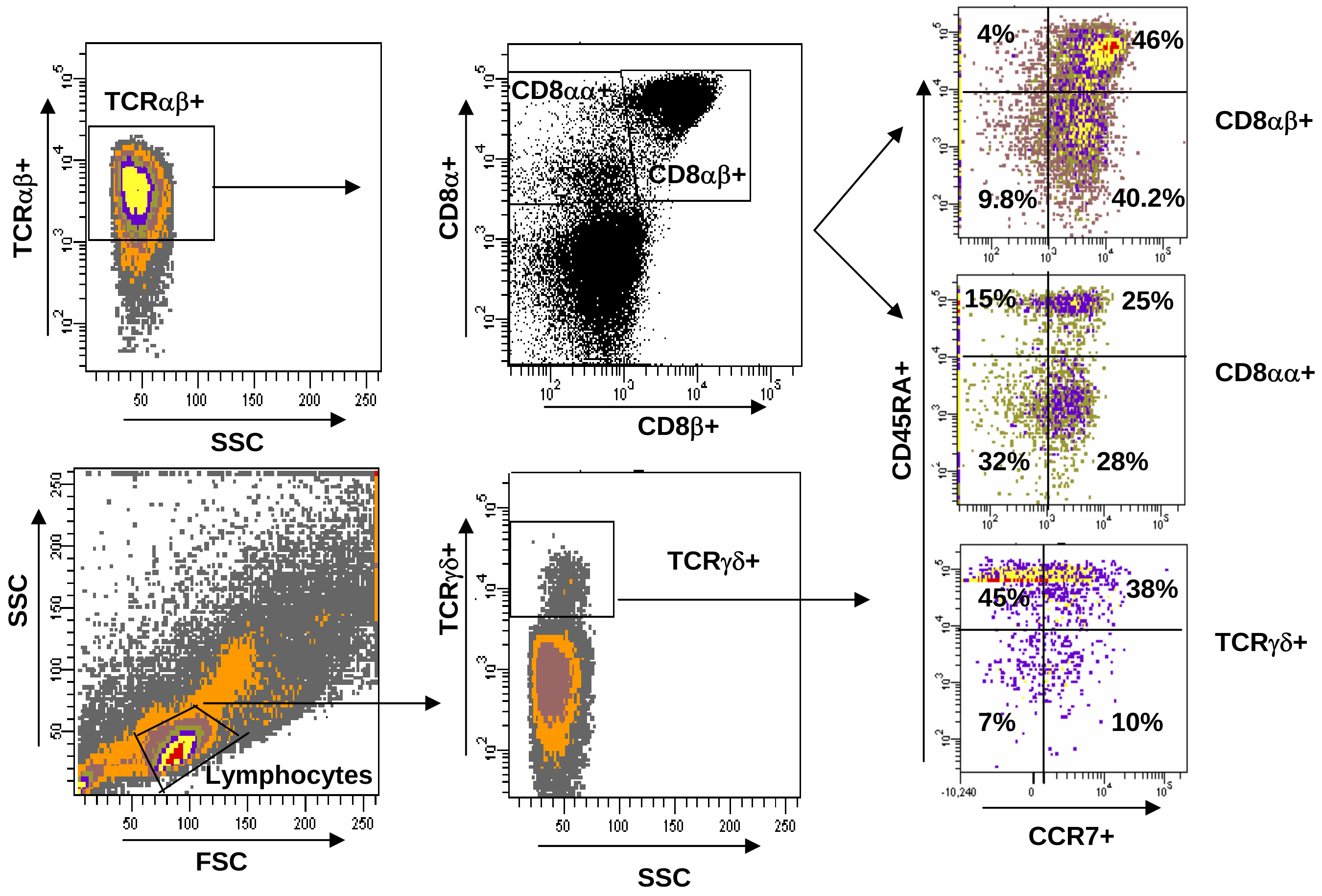

G 2 Expression of CD27 and CD28 on TCRαβ+CD4+CD25+ T-cell subsets

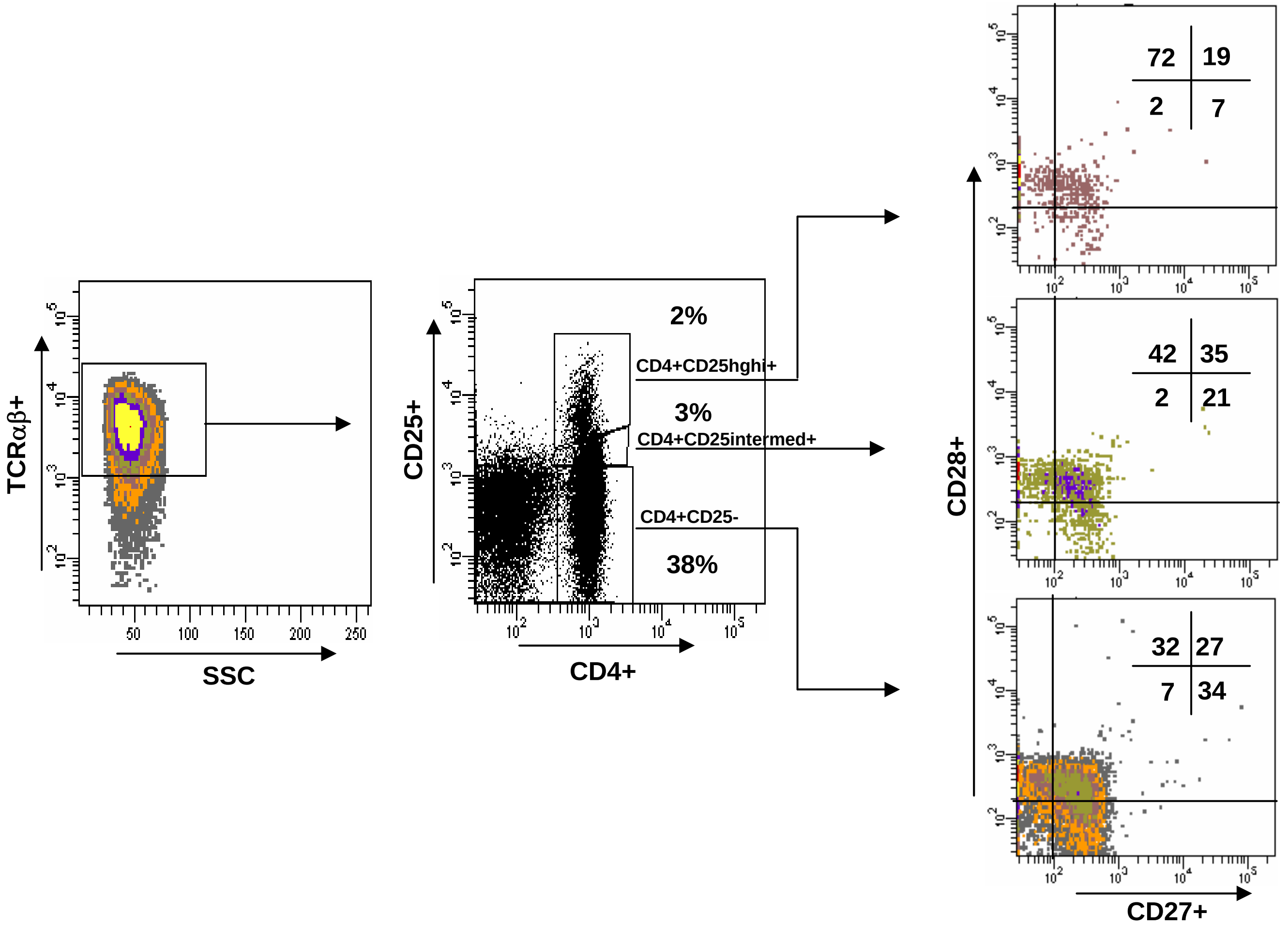

G 3 Expression of CD27 and CD28 on TCRαβ+CD4+CD25+ Foxp3+T-cell subsets

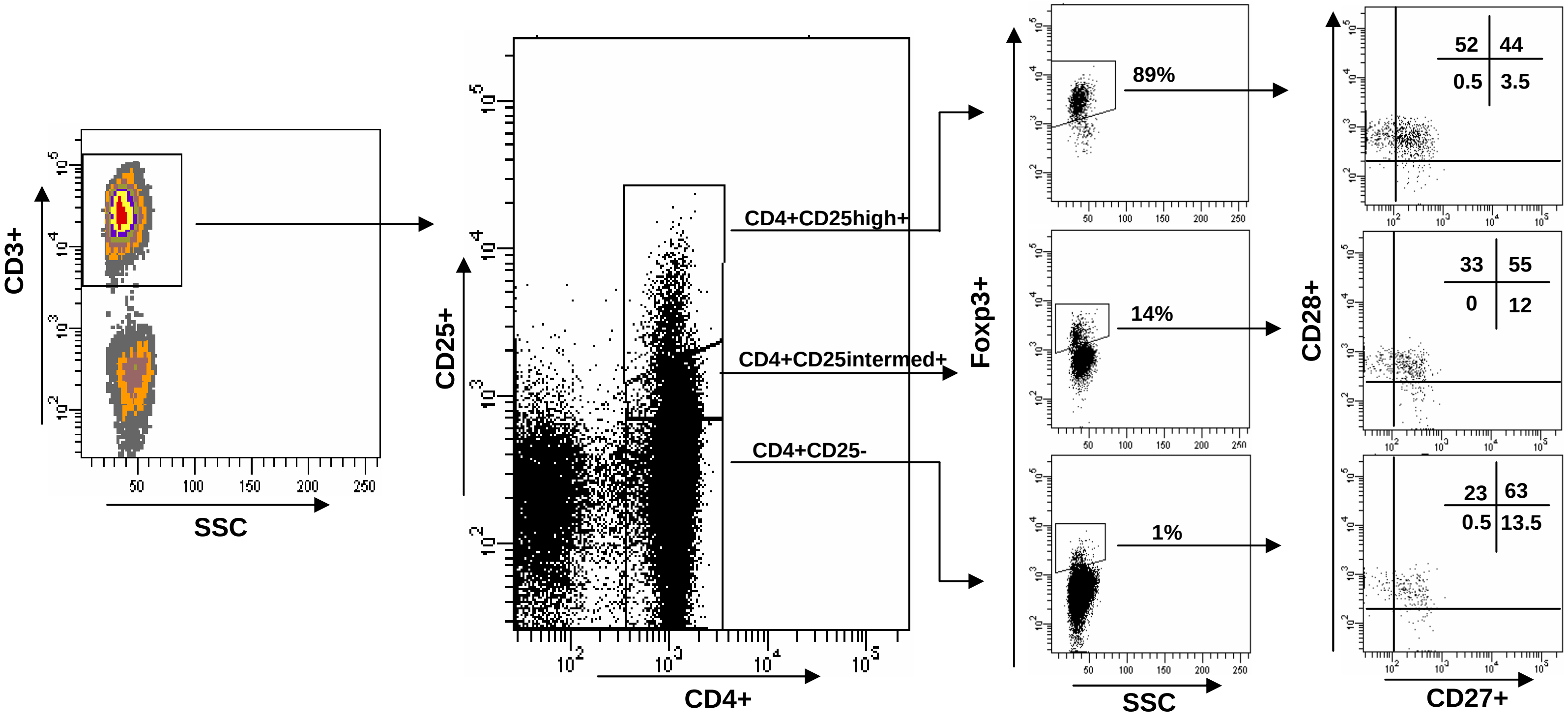

G 4 Expression of IL-7R on TCRαβ+CD4+CD25-CD107a+ T-cells

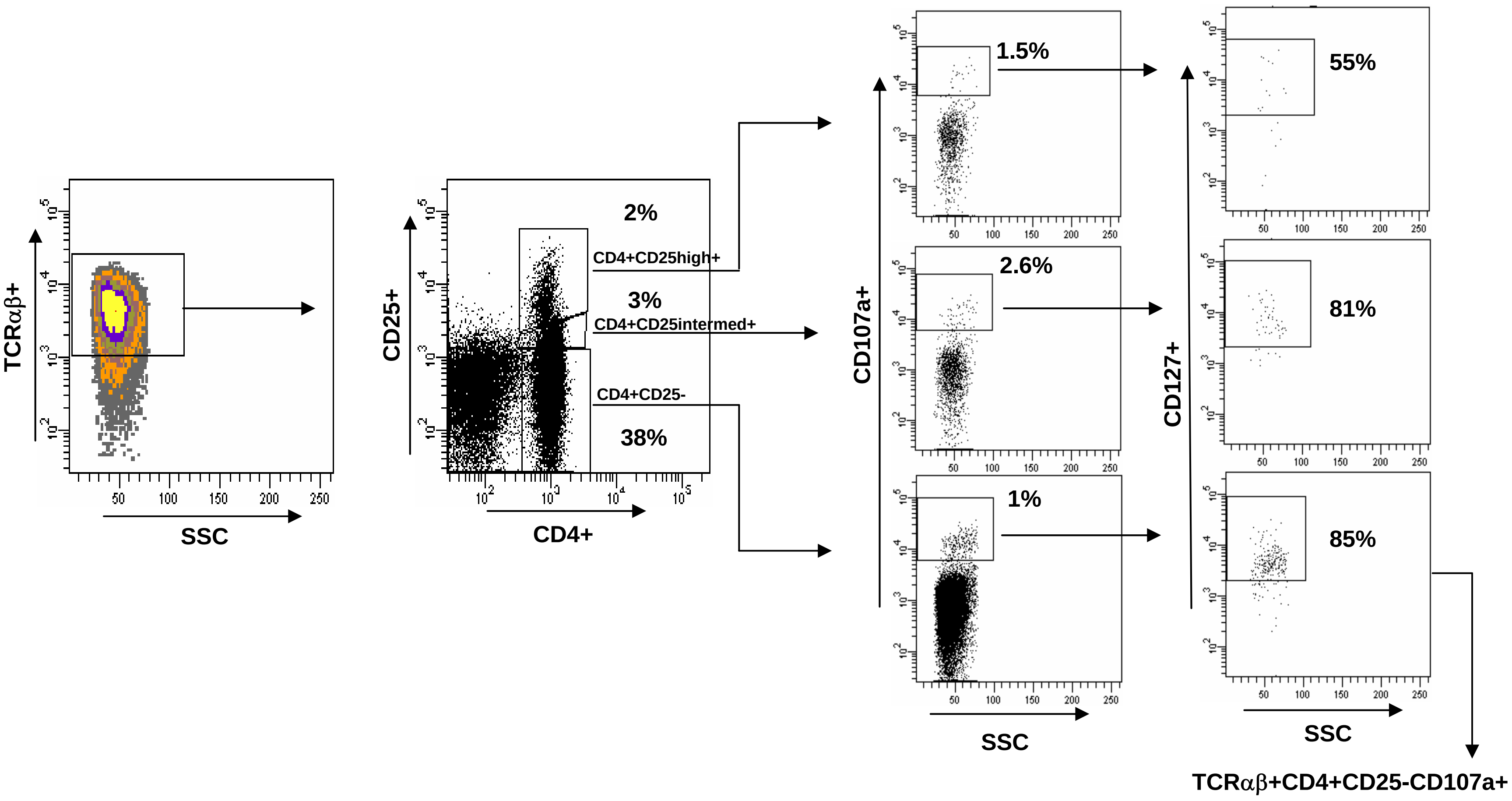

G 5 Expression of IL-7R on the TCRαβ+CD4+CD25int+ T-cell subset

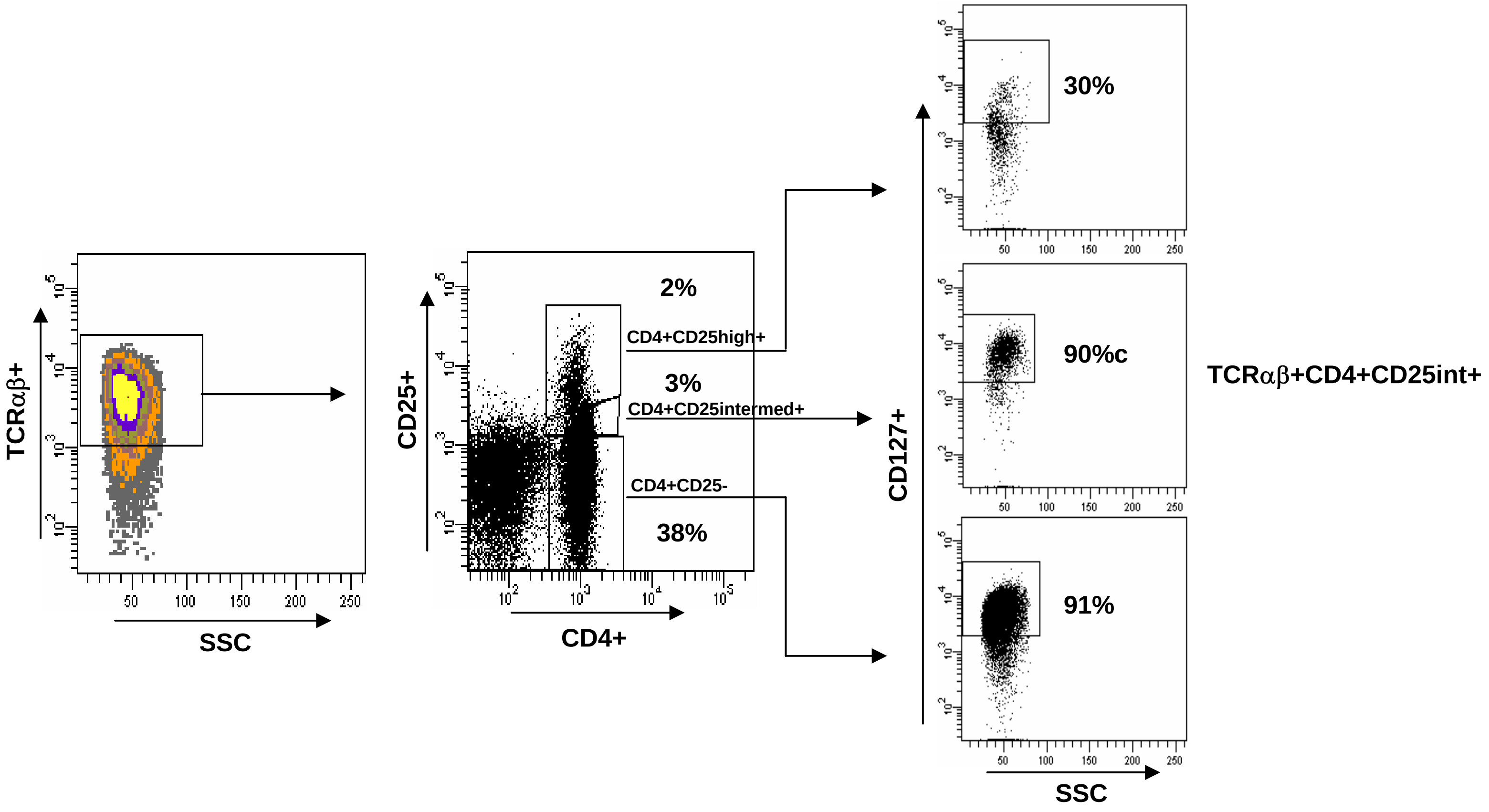

Supplement: Figure S1 — Detailed gating strategies for T-cell subsets, including Tregs. (0.07 MB PDF) [file pone.0006534.s001.pdf]
