# Supplementary material for: Increased Numbers of IL-7 Receptor Molecules on CD4+CD25−CD107a+ T-Cells in Patients with Autoimmune Diseases Affecting the Central Nervous System
Source: PLoS One. 2009 Aug 6;4(8):e6534. doi: 10.1371/journal.pone.0006534 (PMC2717329; doi:10.1371/journal.pone.0006534)
Supplement: Figure S2 — Paradigm of IL-7R (CD127) enumeration on immune cells. A. Cells from the lymphogate were subjected to further gating (n = 59 different subsets). The anti-IL-7 receptor alpha chain monoclonal mAb (clone R34.34) labeled with a single APC molecule was used to define CD127 antibody binding sites, since the APC fluorescence intensity can be compared to the fluorescence intensity associated with the defined number of APC molecules on beads (middle panel) with different APC molecules (top panel, right). A calibration plot was established using Quickcal software V2.3 (Bangs Laboratories, Inc. USA) using the median channel fluorescence intensity values for each bead population loaded with a distinct number of APC molecules. All PBMC samples were run with the same instrumentation settings. Antibody binding capacity values per cell were determined using the median value of CD127 (in APC) of each immune cell subset using Quick Cal soft using the beads calibration plot. B. Although the number of CD127+ T-cells may be different, e.g. lower in individuals with autoimmune disease, the expression of CD127 molecules on single cells may be higher. (0.05 MB PDF) [file pone.0006534.s002.pdf]

Supplementary Figure S 2

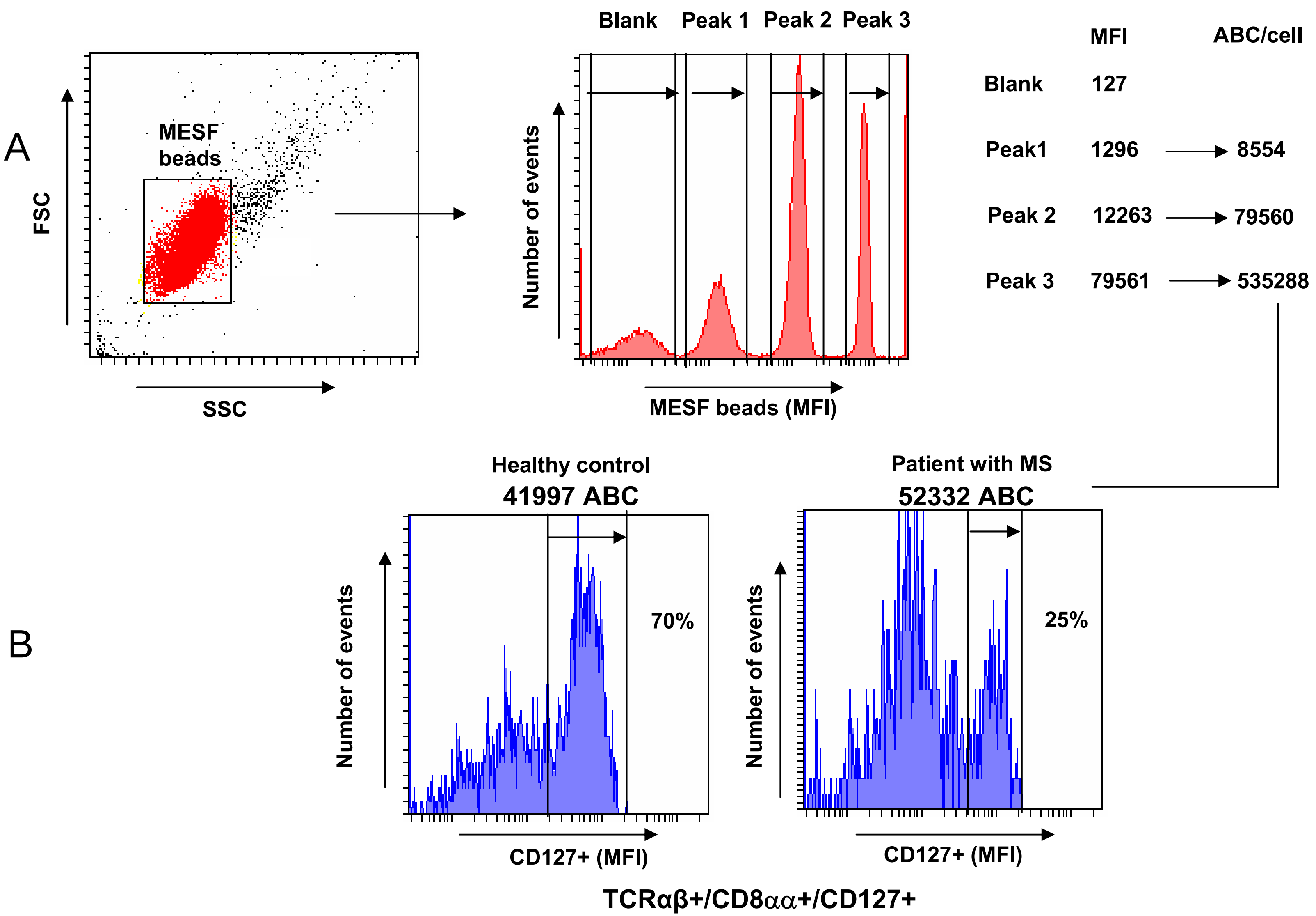

MESF- Molecules of equivalent soluble fluorochrome units

ABC – Antibody binding capacity

MFI – Median fluorescence intensity
