# Supplementary material for: Increased Numbers of IL-7 Receptor Molecules on CD4+CD25−CD107a+ T-Cells in Patients with Autoimmune Diseases Affecting the Central Nervous System
Source: PLoS One. 2009 Aug 6;4(8):e6534. doi: 10.1371/journal.pone.0006534 (PMC2717329; doi:10.1371/journal.pone.0006534)
Supplement: Table S1 — Compilation of monoclonal antibodies and immune cell subsets. (0.03 MB PDF) [file pone.0006534.s003.pdf]

Supplementary Tables S1

S1.1 List of monoclonal antibodies

Antibody	Clone	Company	Isotype control
CD25-PE	2A3	BD Biosciences, San Jose, USA	Mouse IgG1 k chain
CD45RA-ECD	2H4	Immunotech, Marseille, France	Mouse IgG1
TCRαβ-Percp	WT31	BD Biosciences (Custom), San Jose, USA	Mouse IgG1
CCR7-PECy7	3D12	BD Pharmingen, San Jose, USA	Rat IgG2a, k
CD127-APC	R34.34	Beckman Coulter (Custom), Miami, USA	Mouse IgG1
CD107a-APCAlexa 700	H4A3	Beckman Coulter (Custom), Miami, USA	Mouse IgG1
CD8α-APC Cy7	SFC121Thy2D3(T8)	Beckman Coulter (Custom), Miami, USA	Mouse IgG1
CD4-Pacific blue	SFC1 12T4D11	Beckman Coulter (Custom), Miami, USA	Mouse IgG1
CD27 unconjugated	1A4CD27	Beckman Coulter (Custom), Miami, USA	Mouse IgG1
CD28-Amcyan green	CD28.2	BD Biosciences (Custom), San Jose, USA	Mouse IgG1
CD8β-FITC	2ST8.5H7	Beckman Coulter (Custom), Miami, USA	Mouse IgG2a
CD16- PE	3G8	BD Pharmingen, San Jose, USA	Mouse IgG1 k chain
CD56- PE	B159	BD Pharmingen, San Jose, USA	Mouse IgG1 k chain
TCRγδ-PECy5.5	IMMU510	Beckman Coulter (Custom), Miami, USA	Mouse IgG1
FoxP3- Alexa 488	259D	Biolegend, San Diego, USA	Mouse IgG1,k
CD3-ECD	UCHT1	Immunotech, Marseille, France	Mouse IgG1
CD19-FITC	SJ25C1	BD Biosciences, San Jose, USA	Mouse IgG1 k chain
CD14-PE Cy5.5	61D3	Abcam, Cambridge, UK	Mouse IgG1
p-STAT5- Alexa 488	47	BD Biosciences, San Jose, USA	Mouse IgG1
CD4-PECy5	13B8.2	Immunotech, Marseille, France	Mouse IgG1
CD8-PECy7	T8 clone	Beckman Coulter, Fullerton, USA	Mouse IgG1
CD19-PE	SJ25C1	BD Biosciences, San Jose, USA	Mouse IgG1 k chain

S1.2 Combination of monoclonal antibodies for immune cell marker analysis in flow cytometry

Panel 1	Panel 2	Panel 3	Panel 4
CD19-FITC	CD8β - FITC	Foxp3-Alexa 488	pSTAT5 - Alexa 488
CD25-PE	CD16/56 - PE	CD25-PE	CD19 - PE
CD45RA -ECD	CD45RA -ECD	CD3- ECD	CD3 - ECD
TCRαβ-PerCP	TCRab-PerCP	CD127- APC	CD4 - PECy5
CD14 -PE Cy5.5	TCRγδ -PE Cy5.5	CD8α-APC Cy7	CD8α - PECy7
CCR7- PECy7	CCR7- PECy7	CD4- Pacific blue	
CD127- APC	CD127- APC	CD27 - Pacific Orange	
CD107a- APC Alexa700	CD107a- APC Alexa700	CD28- Amcyan green	
CD8α- APC Cy7	CD8α- APC Cy7		
CD4- Pacific blue	CD4- Pacific blue		
CD27 - Pacific Orange	CD27 - Pacific Orange		
CD28- Amcyan green	CD28- Amcyan green		

S1.3 187 T-cell subsets analyzed by using High content Immune flow-cytometry

**Panel 1** immune phenotypes analyzed		**Panel 2**	
TCRαβ+	TCRαβ+CD4+CD8+CD127+	TCRαβ+	TCRαβ+CD8αα+CD27-CD28-
TCRαβ+CD4+	TCRαβ+CD4-CD8-CD25+	TCRαβ-	TCRαβ+CD8αβ+CD45RA+CCR7+CD27+CD28+
TCRαβ+CD8+	TCRαβ+CD4-CD8- CD45RA+CCR7+	TCRαβ+CD8αβ+	TCRαβ+CD8αβ+CD45RA+CCR7+CD27+CD28-
TCRαβ+CD4+CD25high+	TCRαβ+CD4-CD8-CD45RA-CCR7+	TCRαβ+CD8αα+	TCRαβ+CD8αβ+CD45RA+CCR7+CD27-CD28+
TCRαβ+CD4+CD25int+	TCRαβ+CD4-CD8-CD45RA-CCR7-	TCRαβ-CD16+CD56+ (NK cells)	TCRαβ+CD8αβ+CD45RA+CCR7+CD27-CD28-
TCRαβ+CD4+CD127	TCRαβ+CD4-CD8-CD45RA+CCR7-	TCRαβ+CD16+CD56+ (NK-T cells)	TCRαβ+CD8αα+CD45RA+CCR7+CD27+CD28+
TCRαβ+CD8+CD127+	TCRαβ+CD4-CD8- CD45RA+CCR7+CD127+	TCRγδ+	TCRαβ+CD8αα+CD45RA+CCR7+CD27+CD28-
TCRαβ+CD4+CD25high+127+	TCRαβ+CD4-CD8-CD45RA-CCR7+CD127+	TCRαβ+CD8αβ+127+	TCRαβ+CD8αα+CD45RA+CCR7+CD27-CD28+
TCRαβ+CD4+CD25int+127+	TCRαβ+CD4-CD8-CD45RA-CCR7-CD127+	TCRαβ+CD8αα+127+	TCRαβ+CD8αα+CD45RA+CCR7+CD27-CD28-
TCRαβ+CD4+CD25neg+127+	TCRαβ+CD4-CD8-CD45RA+CCR7-CD127+	TCRαβ-CD16+CD56+127+	TCRαβ+CD8αβ+CD45RA-CCR7+CD27+CD28+
TCRαβ+CD4+CD25high+CD45RA+CCR7+127+	TCRαβ+CD4-CD8-CD107a+	TCRαβ+CD16+CD56+127+	TCRαβ+CD8αβ+CD45RA-CCR7+CD27+CD28-
TCRαβ+CD4+CD25int+CD45RA+CCR7+127+	TCRαβ+CD8α+CD4+	TCRγδ+127+	TCRαβ+CD8αβ+CD45RA-CCR7+CD27-CD28+
TCRαβ+CD4+CD25neg+CD45RA+CCR7+127+	TCRαβ+CD8α+CD4+CD127+	TCRαβ+CD8αβ+CD45RA+CCR7+	TCRαβ+CD8αβ+CD45RA-CCR7+CD27-CD28-
TCRαβ+CD4+CD25high+CD45RA-CCR7+127+	TCRαβ+CD8α+CD4-CD127+	TCRαβ+CD8αα+CD45RA+CCR7+	TCRαβ+CD8αα+CD45RA-CCR7+CD27+CD28+
TCRαβ+CD4+CD25int+CD45RA-CCR7+127+	CD14+	TCRγδ+CD45RA+CCR7+	TCRαβ+CD8αα+CD45RA-CCR7+CD27+CD28-
TCRαβ+CD4+CD25neg+CD45RA-CCR7+127+	CD14+CD127+	TCRαβ+CD8αβ+CD45RA-CCR7+	TCRαβ+CD8αα+CD45RA-CCR7+CD27-CD28+
TCRαβ+CD4+CD25high+CD45RA-CCR7-127+	CD14+CD25+	TCRαβ+CD8αα+CD45RA-CCR7+	TCRαβ+CD8αα+CD45RA-CCR7+CD27-CD28-
TCRαβ+CD4+CD25int+CD45RA-CCR7-127+	CD19+	TCRγδ+CD45RA-CCR7+	TCRαβ+CD8αβ+CD45RA-CCR7-CD27+CD28+
TCRαβ+CD4+CD25neg+CD45RA-CCR7-127+	CD19+CD127+	TCRαβ+CD8αβ+CD45RA-CCR7-	TCRαβ+CD8αβ+CD45RA-CCR7-CD27+CD28-
TCRαβ+CD4+CD25high+CD45RA+CCR7-127+	CD19+CD25+	TCRαβ+CD8αα+CD45RA-CCR7-	TCRαβ+CD8αβ+CD45RA-CCR7-CD27-CD28+
TCRαβ+CD4+CD25int+CD45RA+CCR7-127+		TCRγδ+CD45RA-CCR7-	TCRαβ+CD8αβ+CD45RA-CCR7-CD27-CD28-
TCRαβ+CD4+CD25neg+CD45RA+CCR7-127+		TCRαβ+CD8αβ+CD45RA+CCR7-	TCRαβ+CD8αα+CD45RA-CCR7-CD27+CD28+
TCRαβ+CD4+CD25high+CD107a+		TCRαβ+CD8αα+CD45RA+CCR7-	TCRαβ+CD8αα+CD45RA-CCR7-CD27+CD28-
TCRαβ+CD4+CD25int+CD107a+		TCRγδ+CD45RA+CCR7-	TCRαβ+CD8αα+CD45RA-CCR7-CD27-CD28+
TCRαβ+CD4+CD25neg+CD107a+		TCRαβ+CD8αβ+CD45RA+CCR7+127+	TCRαβ+CD8αα+CD45RA-CCR7-CD27-CD28-
TCRαβ+CD4+CD25high+CD107a+127+		TCRαβ+CD8αα+CD45RA+CCR7+127+	TCRαβ+CD8αβ+CD45RA+CCR7-CD27+CD28+
TCRαβ+CD4+CD25int+CD107a+127+		TCRαβ+CD45RA+CCR7+127+	TCRαβ+CD8αβ+CD45RA+CCR7-CD27+CD28-
TCRαβ+CD4+CD25neg+CD107a+127+		TCRαβ+CD8αβ+CD45RA-CCR7++127+	TCRαβ+CD8αβ+CD45RA+CCR7-CD27-CD28+
TCRαβ+CD4-CD8-		TCRαβ+CD8αα+CD45RA-CCR7+127+	TCRαβ+CD8αβ+CD45RA+CCR7-CD27-CD28-
TCRαβ+CD4-CD8-CD127+		TCRγδ+CD45RA-CCR7+127+	TCRαβ+CD8αα+CD45RA+CCR7-CD27+CD28+
TCRαβ+CD4-CD8-CD127+CD25+		TCRαβ+CD8αβ+CD45RA-CCR7-127+	TCRαβ+CD8αα+CD45RA+CCR7-CD27+CD28-
TCRαβ+CD4-CD8-CD127-CD25+		TCRαβ+CD8αα+CD45RA-CCR7-127+	TCRαβ+CD8αα+CD45RA+CCR7-CD27-CD28+
TCRαβ+CD4+CD8+		TCRγδ+CD45RA-CCR7-127+	TCRαβ+CD8αα+CD45RA+CCR7-CD27-CD28-
		TCRαβ+CD8αβ+CD45RA+CCR7-127+	TCRαβ+CD8αβ+107a
		TCRαβ+CD8αα+CD45RA+CCR7-127+	TCRαβ+CD8αα+107a
		TCRγδ+CD45RA+CCR7-127+	TCRγδ+ 107a
		TCRαβ+CD8αβ+CD27+CD28+	TCRαβ-CD16+CD56+CD107a
		TCRαβ+CD8αβ+CD27+CD28-	TCRαβ+CD16+CD56+ CD107a+
		TCRαβ+CD8αβ+CD27-CD28+	TCRαβ+CD8αβ+CD107a+127+
		TCRαβ+CD8αβ+CD27-CD28-	TCRαβ+CD8αα+CD107a+127+
		TCRαβ+CD8αα+CD27+CD28+	TCRαβ+TCRgd+ 107a+127+
		TCRαβ+CD8αα+CD27+CD28-	TCRαβ-CD16+CD56+CD107a+127+
		TCRαβ+CD8αα+CD27-CD28+	TCRαβ+CD16+CD56+CD107a+127+

(Continued on next page for panel 3 and 4)

Panel 3	Panel 4
CD3+	
CD3+CD4+CD25high+(parent)	
CD3+CD4+CD25int+	CD3+
CD3+CD4+CD25high+Foxp3+ Parent	CD19+
CD3+CD4+CD25high+Foxp3+Total	CD3+CD4+
CD3+CD4+CD25int+Foxp3+	CD3+CD8+
CD3+CD4+CD25neg+Foxp3+	CD3+CD4+pStat5+
CD3+CD4+CD25high+Foxp3+CD127+	CD3+CD8+pStat5+
CD3+CD4+CD25int+Foxp3+CD127+	CD19+pStat5+
CD3+CD4+CD25neg+Foxp3+CD127+	
CD3+CD4+CD25high+CD127+	
CD3+CD4+CD25int+CD127+	
CD3+CD4+CD25neg+CD127+	
CD3+CD25high+Foxp3+CD27+CD28+	
CD3+CD25high+Foxp3+CD27+CD28-	
CD3+CD25high+Foxp3+CD27-CD28+	
CD3+CD25high+Foxp3+CD27-CD28-	
CD3+CD25int+Foxp3+CD27+CD28+	
CD3+CD25int+Foxp3+CD27+CD28-	
CD3+CD25int+Foxp3+CD27-CD28+	
CD3+CD25int+Foxp3+CD27-CD28-	
CD3+CD25neg+Foxp3+CD27+CD28+	
CD3+CD25neg+Foxp3+CD27+CD28-	
CD3+CD25neg+Foxp3+CD27-CD28+	
CD3+CD25neg+Foxp3+CD27-CD28-	
CD3+CD25high+CD27+CD28+	
CD3+CD25high+CD27+CD28-	
CD3+CD25high+CD27-CD28+	
CD3+CD25high+CD27-CD28-	
CD3+CD25int+CD27+CD28+	
CD3+CD25int+CD27+CD28-	
CD3+CD25int+CD27-CD28+	
CD3+CD25int+CD27-CD28-	
CD3+CD25neg+CD27+CD28+	
CD3+CD25neg+CD27+CD28-	
CD3+CD25neg+CD27-CD28+	
CD3+CD25neg+CD27-CD28-	
CD3+CD8+CD25+	
CD3+CD8+CD25+Foxp3+	
CD3+CD8+CD25+Foxp3+CD127+	
CD3+CD8+CD25+Foxp3+CD27+CD28+	
CD3+CD8+CD25+Foxp3+CD27+CD28-	
CD3+CD8+CD25+Foxp3+CD27-CD28+	
CD3+CD8+CD25+Foxp3+CD27-CD28-	

S1.4 IL-7 receptor density on 59T-cell subsets

Panel 1	Panel 2	Panel 3
TCRαβ+CD4+CD127+	TCRαβ+CD8αβ+CD127+	CD3+CD4+CD25high+Foxp3+CD127+
TCRαβ+CD8+CD127+	TCRαβ+CD8αα+CD127+	CD3+CD4+CD25int+Foxp3+CD127+
TCRαβ+CD4+CD25high+127+	TCRαβ-CD16+CD56+CD127+	CD3+CD4+CD25neg+Foxp3+CD127+
TCRαβ+CD4+CD25int+127+	TCRαβ+CD16+CD56+CD127+	CD3+CD4+CD25high+CD127+
TCRαβ+CD4+CD25neg+127+	TCRγδ+CD127+	CD3+CD4+CD25int+CD127+
TCRαβ+CD4+CD25high+CD45RA+CCR7+CD127+	TCRαβ+CD8αβ+CD45RA+CCR7+CD127+	CD3+CD4+CD25neg+CD127+
TCRαβ+CD4+CD25int+CD45RA+CCR7+CD127+	TCRαβ+CD8αα+CD45RA+CCR7+CD127+	CD3+CD8+CD25+Foxp3+CD127+
TCRαβ+CD4+CD25neg+CD45RA+CCR7+CD127+	TCRγδ+CD45RA+CCR7+CD127+	
TCRαβ+CD4+CD25high+CD45RA-CCR7+CD127+	TCRαβ+CD8αβ+CD45RA-CCR7+CD127+	
TCRαβ+CD4+CD25int+CD45RA-CCR7+CD127+	TCRαβ+CD8αα+CD45RA-CCR7+CD127+	
TCRαβ+CD4+CD25neg+CD45RA-CCR7+CD127+	TCRγδ+CD45RA-CCR7+CD127+	
TCRαβ+CD4+CD25high+CD45RA-CCR7-CD127+	TCRαβ+CD8αβ+CD45RA-CCR7-CD127+	
TCRαβ+CD4+CD25int+CD45RA-CCR7-CD127+	TCRαβ+CD8αα+CD45RA-CCR7-CD127+	
TCRαβ+CD4+CD25neg+CD45RA-CCR7-CD127+	TCRγδ+CD45RA-CCR7-CD127+	
TCRαβ+CD4+CD25high+CD45RA+CCR7-CD127+	TCRαβ+CD8αβ+CD45RA+CCR7-CD127+	
TCRαβ+CD4+CD25int+CD45RA+CCR7-CD127+	TCRαβ+CD8αα+CD45RA+CCR7-CD127+	
TCRαβ+CD4+CD25neg+CD45RA+CCR-CD127+	TCRγδ+CD45RA+CCR7-CD127+	
TCRαβ+CD4+CD25high+CD107a+CD127+	TCRαβ+CD8αβ+CD107a+CD127+	
TCRαβ+CD4+CD25int+Cd107a+CD127+	TCRαβ+CD8αα+CD107a+CD127+	
TCRαβ+CD4+CD25neg+CD107a+CD127+	TCRγδ+CD107a+CD127+	
TCRαβ+CD8α+CD4+CD127+	TCRαβ-CD16+CD56+CD107a+CD127+	
TCRαβ+CD8α+CD4-CD127+	TCRαβ+CD16+CD56+CD107a+CD127+	
TCRαβ+CD4-CD8-CD127+		
TCRαβ+CD4+CD8+CD127+		
TCRαβ+CD4-CD8-CD45RA+CCR7+CD127+		
TCRαβ+CD4-CD8-CD45RA-CCR7+CD127+		
TCRαβ+CD4-CD8-CD45RA-CCR7-CD127+		
TCRαβ+CD4-CD8-CD45RA+CCR7-CD127+		
CD14+CD127		
CD19+CD127		
