# Supplementary material for: Increased Numbers of IL-7 Receptor Molecules on CD4+CD25−CD107a+ T-Cells in Patients with Autoimmune Diseases Affecting the Central Nervous System
Source: PLoS One. 2009 Aug 6;4(8):e6534. doi: 10.1371/journal.pone.0006534 (PMC2717329; doi:10.1371/journal.pone.0006534)
Supplement: Table S2 — Mean and Median values of IL-7 Receptor densities on T-cell subsets (0.08 MB PDF) [file pone.0006534.s004.pdf]

Supplementary Table S2

S2.1 IL-7R density on T- cell subsets from PBMCs obtained from Healthy donors

Subject ID	Mean	Median	S.D.
CD14+CD127+	18452	18938	4437
CD19+CD127	20999	20958	6151
TCRαβ+CD4-CD8-CD127+	35129	33788	9699
TCRαβ+CD4+CD8+CD127+	40296	41079	11481
TCRαβ+CD4-CD8- CD45RA+CCR7+CD127+	30541	27601	7264
TCRαβ+CD4-CD8-CD45RA-CCR7+CD127+	37060	34080	10040
TCRαβ+CD4-CD8-CD45RA-CCR7-CD127+	38853	36249	11871
TCRαβ+CD4-CD8-CD45RA+CCR7-CD127+	27822	29564	7291
TCRαβ+CD8α+CD4+CD127+	39292	34223	16330
TCRαβ+CD8α+CD4-CD127+	39664	37118	12726
TCRαβ+CD8αβ+CD127+	31567	31285	5983
TCRαβ+CD8αα+CD127+	38445	38054	11935
TCRαβ-CD16+CD56+CD127+	20588	21713	3958
TCRαβ+CD16+CD56+CD127+	28276	27979	9527
TCRγδ+CD127+	30943	31230	6170
TCRαβ+CD8αβ+CD45RA+CCR7+CD127+	29616	31396	4972
TCRαβ+CD8αα+CD45RA+CCR7+CD127+	28176	28174	6949
TCRγδ+CD45RA+CCR7+CD127+	37225	35140	7678
TCRαβ+CD8αβ+CD45RA-CCR7+CD127+	36047	33820	7112
TCRαβ+CD8αα+CD45RA-CCR7+CD127+	38653	37363	13053
TCRγδ+CD45RA-CCR7+CD127+	38401	35155	14270
TCRαβ+CD8αβ+CD45RA-CCR7-CD127+	33145	32523	7538
TCRαβ+CD8αα+CD45RA-CCR7-CD127+	39928	39907	12313
TCRγδ+CD45RA-CCR7-CD127+	29957	31904	7649
TCRαβ+CD8αβ+CD45RA+CCR7-CD127+	27300	26959	5515
TCRαβ+CD8αα+CD45RA+CCR7-CD127+	31131	29736	8473
TCRγδ+CD45RA+CCR7-CD127+	25297	25859	4698
TCRαβ+CD8αβ+CD107a+127+	40640	35763	18193
TCRαβ+CD8αα+CD107a+127+	26420	26592	10870
TCRγδ+ CD107a+127+	38287	39166	12964
TCRαβ-CD16+CD56+CD107a+127+	34542	28822	15604
TCRαβ+CD16+CD56+CD107a+127+	61228	73371	25416
TCRαβ+CD4+CD127	34771	31842	8833
TCRαβ+CD8+CD127+	30843	28960	6395
TCRαβ+CD4+CD25hi+127+	39864	38695	8892
TCRαβ+CD4+CD25int+127+	44663	44285	11569
TCRαβ+CD4+CD25-127+	33529	32731	6414
TCRαβ+CD4+CD25hi+CD45RA+CCR7+CD127+	31252	29115	7932
TCRαβ+CD4+CD25int+CD45RA+CCR7+CD127+	34336	35164	5717
TCRαβ+CD4+CD25-CD45RA+CCR7+CD127+	30564	29940	4107
TCRαβ+CD4+CD25hi+CD45RA-CCR7+CD127+	42375	40672	9351
TCRαβ+CD4+CD25int+CD45RA-CCR7+CD127+	46608	44624	10997
TCRαβ+CD4+CD25-CD45RA-CCR7+CD127+	36366	34831	7799
TCRαβ+CD4+CD25hi+CD45RA-CCR7-CD127+	44632	43382	10101
TCRαβ+CD4+CD25int+CD45RA-CCR7-CD127+	47334	43444	11311
TCRαβ+CD4+CD25-CD45RA-CCR7-CD127+	36925	33527	8992
TCRαβ+CD4+CD25hi+CD45RA+CCR7-CD127+	30313	30383	8576
TCRαβ+CD4+CD25int+CD45RA+CCR7-CD127+	36712	37202	8343
TCRαβ+CD4+CD25-CD45RA+CCR7-CD127+	28515	28283	3800
TCRαβ+CD4+CD25hi+CD107a+127+	53932	46405	32072
TCRαβ+CD4+CD25int+CD107a+127+	47562	47510	10814
TCRαβ+CD4+CD25-CD107a+127+	28664	28376	4865
TCRαβ+CD25hi+Foxp3+CD127+	24723	24596	5351
TCRαβ+CD25int+Foxp3+CD127+	35946	35617	6284
TCRαβ+CD25neg+Foxp3+CD127+	26288	25448	4484
TCRαβ+CD25hi+CD127+	28243	30401	4999
TCRαβ+CD25int+CD127+	28132	28643	5046
TCRαβ+CD25-CD127+	19937	20139	2885
TCRαβ+CD8+CD25+Foxp3+CD127+	54398	35048	48830
TCRαβ+CD4+CD45RA+CCR7+CD127+	30083	30057	4532
TCRαβ+CD4+CD45RA-CCR7+CD127+	40449	40205	9186
TCRαβ+CD4+CD45RA-CCR7-CD127+	42428	39053	11146
TCRαβ+CD4+CD45RA+CCR7-CD127+	27702	28253	4170

S2.2 IL-7R density on T- cell subsets from PBMCs obtained from patients with RRMS

Subject ID	Mean	Median	S.D.
CD14+CD127+	20218	18220	5141
CD19+CD127	33002	33388	14881
TCRαβ+CD4-CD8-CD127+	42197	38900	12899
TCRαβ+CD4+CD8+CD127+	43108	43847	12143
TCRαβ+CD4-CD8- CD45RA+CCR7+CD127+	33780	36607	11179
TCRαβ+CD4-CD8-CD45RA-CCR7+CD127+	43900	38654	16246
TCRαβ+CD4-CD8-CD45RA-CCR7-CD127+	44729	40595	16597
TCRαβ+CD4-CD8-CD45RA+CCR7-CD127+	31186	30209	9931
TCRαβ+CD8α+CD4+CD127+	38212	36531	13019
TCRαβ+CD8α+CD4-CD127+	41967	45563	15997
TCRαβ+CD8αβ+CD127+	35204	37624	7780
TCRαβ+CD8αα+CD127+	41737	42245	16221
TCRαβ-CD16+CD56+CD127+	23737	25603	5229
TCRαβ+CD16+CD56+CD127+	30674	28515	8205
TCRγδ+CD127+	35592	36498	7744
TCRαβ+CD8αβ+CD45RA+CCR7+CD127+	33205	36481	7329
TCRαβ+CD8αα+CD45RA+CCR7+CD127+	32208	35093	10501
TCRγδ+CD45RA+CCR7+CD127+	33222	32350	8545
TCRαβ+CD8αβ+CD45RA-CCR7+CD127+	41008	44224	9913
TCRαβ+CD8αα+CD45RA-CCR7+CD127+	42173	31544	16406
TCRγδ+CD45RA-CCR7+CD127+	39884	38739	8852
TCRαβ+CD8αβ+CD45RA-CCR7-CD127+	39197	39084	12978
TCRαβ+CD8αα+CD45RA-CCR7-CD127+	44001	45995	15612
TCRγδ+CD45RA-CCR7-CD127+	35978	35185	9175
TCRαβ+CD8αβ+CD45RA+CCR7-CD127+	32030	35019	8003
TCRαβ+CD8αα+CD45RA+CCR7-CD127+	33805	32447	13985
TCRγδ+CD45RA+CCR7-CD127+	28171	29514	7853
TCRαβ+CD8αβ+CD107a+127+	45546	46752	7971
TCRαβ+CD8αα+CD107a+127+	27263	25911	11937
TCRγδ+ CD107a+127+	50977	45498	23023
TCRαβ-CD16+CD56+CD107a+127+	34273	33593	4386
TCRαβ+CD16+CD56+CD107a+127+	49557	39634	29664
TCRαβ+CD4+CD127	39474	39957	7459
TCRαβ+CD8+CD127+	34507	37471	10121
TCRαβ+CD4+CD25hi+127+	47917	46519	9992
TCRαβ+CD4+CD25int+127+	51429	49170	8267
TCRαβ+CD4+CD25-127+	37888	39232	7385
TCRαβ+CD4+CD25hi+CD45RA+CCR7+CD127+	41278	40589	11798
TCRαβ+CD4+CD25int+CD45RA+CCR7+CD127+	41302	39259	9757
TCRαβ+CD4+CD25-CD45RA+CCR7+CD127+	34563	35996	7765
TCRαβ+CD4+CD25hi+CD45RA-CCR7+CD127+	47706	49249	12508
TCRαβ+CD4+CD25int+CD45RA-CCR7+CD127+	52643	52664	7462
TCRαβ+CD4+CD25-CD45RA-CCR7+CD127+	42142	42261	7197
TCRαβ+CD4+CD25hi+CD45RA-CCR7-CD127+	52740	53226	10496
TCRαβ+CD4+CD25int+CD45RA-CCR7-CD127+	55153	53179	7955
TCRαβ+CD4+CD25-CD45RA-CCR7-CD127+	42088	44114	7080
TCRαβ+CD4+CD25hi+CD45RA+CCR7-CD127+	44589	41024	19240
TCRαβ+CD4+CD25int+CD45RA+CCR7-CD127+	45090	43806	12187
TCRαβ+CD4+CD25-CD45RA+CCR7-CD127+	31743	33680	6911
TCRαβ+CD4+CD25hi+CD107a+127+	56037	50791	14895
TCRαβ+CD4+CD25int+CD107a+127+	51587	43144	17078
TCRαβ+CD4+CD25-CD107a+127+	50734	48515	11987
TCRαβ+CD25hi+Foxp3+CD127+	28183	25364	9609
TCRαβ+CD25int+Foxp3+CD127+	48432	49692	8540
TCRαβ+CD25neg+Foxp3+CD127+	35274	35030	5960
TCRαβ+CD25hi+CD127+	41591	40385	9019
TCRαβ+CD25int+CD127+	38903	39237	8143
TCRαβ+CD25-CD127+	25117	24972	4273
TCRαβ+CD8+CD25+Foxp3+CD127+	40157	44353	20639
TCRαβ+CD4+CD45RA+CCR7+CD127+	34889	37642	9304
TCRαβ+CD4+CD45RA-CCR7+CD127+	45729	46103	9041
TCRαβ+CD4+CD45RA-CCR7-CD127+	47995	47258	9540
TCRαβ+CD4+CD45RA+CCR7-CD127+	33987	33921	10504

S2.3 IL-7R density on T- cell subset from PBMCs obtained from patients with SPMS

Subject ID	Mean	Median	S.D.
CD14+CD127+	20280	19434	3002
CD19+CD127	27879	25744	8078
TCRαβ+CD4-CD8-CD127+	38487	36237	6068
TCRαβ+CD4+CD8+CD127+	45505	43375	6313
TCRαβ+CD4-CD8- CD45RA+CCR7+CD127+	29307	28788	6794
TCRαβ+CD4-CD8-CD45RA-CCR7+CD127+	38774	36800	7025
TCRαβ+CD4-CD8-CD45RA-CCR7-CD127+	43224	39405	10605
TCRαβ+CD4-CD8-CD45RA+CCR7-CD127+	30244	31164	6363
TCRαβ+CD8α+CD4+CD127+	45697	44488	10545
TCRαβ+CD8α+CD4-CD127+	41215	40103	9654
TCRαβ+CD8αβ+CD127+	34940	34046	3264
TCRαβ+CD8αα+CD127+	43961	43503	8254
TCRαβ-CD16+CD56+CD127+	22450	22395	4653
TCRαβ+CD16+CD56+CD127+	30515	28340	7842
TCRγδ+CD127+	38874	35997	8157
TCRαβ+CD8αβ+CD45RA+CCR7+CD127+	31638	31912	3508
TCRαβ+CD8αα+CD45RA+CCR7+CD127+	32450	32012	6830
TCRγδ+CD45RA+CCR7+CD127+	35863	35583	6373
TCRαβ+CD8αβ+CD45RA-CCR7+CD127+	39617	38710	4061
TCRαβ+CD8αα+CD45RA-CCR7+CD127+	44932	45272	9985
TCRγδ+CD45RA-CCR7+CD127+	40099	37866	8238
TCRαβ+CD8αβ+CD45RA-CCR7-CD127+	37821	36930	3862
TCRαβ+CD8αα+CD45RA-CCR7-CD127+	46397	44003	9060
TCRγδ+CD45RA-CCR7-CD127+	37153	36138	8454
TCRαβ+CD8αβ+CD45RA+CCR7-CD127+	29375	28516	4307
TCRαβ+CD8αα+CD45RA+CCR7-CD127+	31954	28632	8765
TCRγδ+CD45RA+CCR7-CD127+	28145	28356	4866
TCRαβ+CD8αβ+CD107a+127+	42941	43832	10534
TCRαβ+CD8αα+CD107a+127+	28720	28927	8317
TCRγδ+ CD107a+127+	47663	38413	32498
TCRαβ-CD16+CD56+CD107a+127+	32868	25511	17609
TCRαβ+CD16+CD56+CD107a+127+	39002	35331	13706
TCRαβ+CD4+CD127	38362	39467	4658
TCRαβ+CD8+CD127+	34433	33573	5506
TCRαβ+CD4+CD25hi+127+	37089	35567	8717
TCRαβ+CD4+CD25int+127+	47931	45994	6727
TCRαβ+CD4+CD25-127+	35350	35828	4117
TCRαβ+CD4+CD25hi+CD45RA+CCR7+CD127+	33935	30314	8346
TCRαβ+CD4+CD25int+CD45RA+CCR7+CD127+	37780	38033	3625
TCRαβ+CD4+CD25-CD45RA+CCR7+CD127+	31904	31138	3426
TCRαβ+CD4+CD25hi+CD45RA-CCR7+CD127+	37617	35567	9793
TCRαβ+CD4+CD25int+CD45RA-CCR7+CD127+	50395	51234	7061
TCRαβ+CD4+CD25-CD45RA-CCR7+CD127+	39491	39116	5023
TCRαβ+CD4+CD25hi+CD45RA-CCR7-CD127+	38286	39488	10407
TCRαβ+CD4+CD25int+CD45RA-CCR7-CD127+	51612	51548	7527
TCRαβ+CD4+CD25-CD45RA-CCR7-CD127+	39964	38047	5622
TCRαβ+CD4+CD25hi+CD45RA+CCR7-CD127+	26726	22478	9986
TCRαβ+CD4+CD25int+CD45RA+CCR7-CD127+	38214	38238	6475
TCRαβ+CD4+CD25-CD45RA+CCR7-CD127+	30692	29188	3858
TCRαβ+CD4+CD25hi+CD107a+127+	58023	67340	20637
TCRαβ+CD4+CD25int+CD107a+127+	56370	59367	12481
TCRαβ+CD4+CD25-CD107a+127+	40634	38195	7469
TCRαβ+CD25hi+Foxp3+CD127+	27142	26666	7256
TCRαβ+CD25int+Foxp3+CD127+	41674	41908	8788
TCRαβ+CD25neg+Foxp3+CD127+	33503	31471	6788
TCRαβ+CD25hi+CD127+	37719	34060	10563
TCRαβ+CD25int+CD127+	36747	36379	7226
TCRαβ+CD25-CD127+	25466	25124	5088
TCRαβ+CD8+CD25+Foxp3+CD127+	60729	55787	29846
TCRαβ+CD4+CD45RA+CCR7+CD127+	31172	30495	4414
TCRαβ+CD4+CD45RA-CCR7+CD127+	42844	41718	7307
TCRαβ+CD4+CD45RA-CCR7-CD127+	44221	40149	8568
TCRαβ+CD4+CD45RA+CCR7-CD127+	29946	28120	4240

S2.4 IL-7R density on T- cell subsets from PBMCs obtained from patients with other neurological diseases

Subject ID	Mean	Median	S.D.
CD14+CD127+	20339	19201	2595
CD19+CD127	30364	27264	11457
TCRαβ+CD4-CD8-CD127+	44675	44012	6960
TCRαβ+CD4+CD8+CD127+	43022	43205	16286
TCRαβ+CD4-CD8- CD45RA+CCR7+CD127+	33297	32016	5821
TCRαβ+CD4-CD8-CD45RA-CCR7+CD127+	45295	45386	5320
TCRαβ+CD4-CD8-CD45RA-CCR7-CD127+	50532	46816	10355
TCRαβ+CD4-CD8-CD45RA+CCR7-CD127+	37988	35961	11028
TCRαβ+CD8α+CD4+CD127+	44362	42108	11290
TCRαβ+CD8α+CD4-CD127+	48244	48226	7859
TCRαβ+CD8αβ+CD127+	33946	33364	6389
TCRαβ+CD8αα+CD127+	48186	46860	8251
TCRαβ-CD16+CD56+CD127+	21946	20511	4756
TCRαβ+CD16+CD56+CD127+	27201	26577	7387
TCRγδ+CD127+	38288	34394	7551
TCRαβ+CD8αβ+CD45RA+CCR7+CD127+	31026	30934	5286
TCRαβ+CD8αα+CD45RA+CCR7+CD127+	33165	32454	4588
TCRγδ+CD45RA+CCR7+CD127+	45599	36314	21437
TCRαβ+CD8αβ+CD45RA-CCR7+CD127+	40790	41356	7543
TCRαβ+CD8αα+CD45RA-CCR7+CD127+	48562	48915	9009
TCRγδ+CD45RA-CCR7+CD127+	42302	43351	9872
TCRαβ+CD8αβ+CD45RA-CCR7-CD127+	38572	40230	8403
TCRαβ+CD8αα+CD45RA-CCR7-CD127+	50700	50047	8861
TCRγδ+CD45RA-CCR7-CD127+	37727	35367	7187
TCRαβ+CD8αβ+CD45RA+CCR7-CD127+	29606	29053	6483
TCRαβ+CD8αα+CD45RA+CCR7-CD127+	36569	33573	7914
TCRγδ+CD45RA+CCR7-CD127+	29400	29844	6585
TCRαβ+CD8αβ+CD107a+127+	43030	39215	23012
TCRαβ+CD8αα+CD107a+127+	37473	37807	11331
TCRγδ+ CD107a+127+	38374	35487	14646
TCRαβ-CD16+CD56+CD107a+127+	30367	26044	11666
TCRαβ+CD16+CD56+CD107a+127+	55587	52834	21770
TCRαβ+CD4+CD127	39806	39757	7661
TCRαβ+CD8+CD127+	34912	35050	7495
TCRαβ+CD4+CD25hi+127+	46291	45030	6293
TCRαβ+CD4+CD25int+127+	51222	49623	6932
TCRαβ+CD4+CD25-127+	37469	37230	5998
TCRαβ+CD4+CD25hi+CD45RA+CCR7+CD127+	38021	36493	9072
TCRαβ+CD4+CD25int+CD45RA+CCR7+CD127+	42366	42297	5176
TCRαβ+CD4+CD25-CD45RA+CCR7+CD127+	34717	34682	4873
TCRαβ+CD4+CD25hi+CD45RA-CCR7+CD127+	45323	44804	8279
TCRαβ+CD4+CD25int+CD45RA-CCR7+CD127+	52219	50477	7382
TCRαβ+CD4+CD25-CD45RA-CCR7+CD127+	41393	39897	6804
TCRαβ+CD4+CD25hi+CD45RA-CCR7-CD127+	51612	50855	7128
TCRαβ+CD4+CD25int+CD45RA-CCR7-CD127+	54309	52236	6954
TCRαβ+CD4+CD25-CD45RA-CCR7-CD127+	42037	41713	4623
TCRαβ+CD4+CD25hi+CD45RA+CCR7-CD127+	34935	36723	13010
TCRαβ+CD4+CD25int+CD45RA+CCR7-CD127+	45109	44376	7379
TCRαβ+CD4+CD25-CD45RA+CCR7-CD127+	32690	33418	4192
TCRαβ+CD4+CD25hi+CD107a+127+	63035	63618	27826
TCRαβ+CD4+CD25int+CD107a+127+	55256	54884	5983
TCRαβ+CD4+CD25-CD107a+127+	36631	33692	10849
TCRαβ+CD25hi+Foxp3+CD127+	23020	19296	8593
TCRαβ+CD25int+Foxp3+CD127+	36415	31343	14350
TCRαβ+CD25neg+Foxp3+CD127+	32034	27563	7912
TCRαβ+CD25hi+CD127+	32980	30437	11934
TCRαβ+CD25int+CD127+	33120	32176	9602
TCRαβ+CD25-CD127+	22291	20235	6719
TCRαβ+CD8+CD25+Foxp3+CD127+	32441	32704	14719
TCRαβ+CD4+CD45RA+CCR7+CD127+	34001	34865	4941
TCRαβ+CD4+CD45RA-CCR7+CD127+	44499	42632	7380
TCRαβ+CD4+CD45RA-CCR7-CD127+	46031	47238	6206
TCRαβ+CD4+CD45RA+CCR7-CD127+	33270	32520	7144
