# Supplementary material for: Increased Numbers of IL-7 Receptor Molecules on CD4+CD25−CD107a+ T-Cells in Patients with Autoimmune Diseases Affecting the Central Nervous System
Source: PLoS One. 2009 Aug 6;4(8):e6534. doi: 10.1371/journal.pone.0006534 (PMC2717329; doi:10.1371/journal.pone.0006534)
Supplement: Table S3 — Number of events (min-max) for TCRαβ+CD4+CD25−CD107a+CD127+ and TCRαβ+CD4+CD25intermed+CD127+ T-cell subsets (0.02 MB PDF) [file pone.0006534.s005.pdf]

Supplementary Table 3

Number of events (min-max) for TCRαβ+CD4+CD25-CD107a+CD127+ and TCRαβ+CD4+CD25intermed+CD127+ T-cell subsets

Group	CD127events+ Freq. of TCRαβ+CD4+CD25-CD107a+	CD127events+ Freq. of TCRαβ+CD4+CD25intermed+ T-cells
HC	2-210 (36)	2286-10882 (6293)
RRMS	6-37 (22)	2086-7643 (4773)
SPMS	4-65 (24)	1917-15733 (5861)
OND	2-42 (15)	1062-9537 (4648)

HC- Healthy Control

SPMS – Secondary Progressive Multiple Sclerosis

RRMS – Relapse and Remittent Multiple Sclerosis

OND- Other Neurological Diseases
